# Translocation-induced enhancer rewiring reveals cryptic oncogenic circuits in multiple myeloma

**DOI:** 10.1126/sciadv.aeg9610

**Published:** 2026-10-07

**Authors:** Enrun Zheng, Qinghua Li, Xuelin Dou, Yiping Cao, Chao Ren, Feiya Suo, Lin Lin, Yan Zhao, Zhiwei Liao, Yang Liu, Zesen Shang, Sudun Guan, Chengying Zhang, Mengyu Yang, Pengyu Xiang, Yakun Chen, Xinhua Liu, Yueyun Lai, Zhiqiang Liu, Adkhamjon Odilovich Abdullayev, Lin He, Xiaochen Bo, Yongfeng Shang, Jichuan Wang, Jin Lu, Hebing Chen, Luyang Sun

**Affiliations:** ^1^ Department of Biochemistry and Molecular Biology, School of Basic Medical Sciences, Peking University International Cancer Institute, State Key Laboratory of Natural and Biomimetic Drugs, Peking University Health Science Center, Beijing 100191, China.; ^2^ Peking University Institute of Hematology, Collaborative Innovation Center of Hematology, Peking University People’s Hospital, Beijing 100044, China.; ^3^ Academy of Military Medical Sciences, Beijing 100850, China.; ^4^ Department of Integration of Chinese and Western Medicine, School of Basic Medical Sciences, Peking University Health Science Center, Beijing 100191, China.; ^5^ Department of Orthopedics, Peking University Third Hospital, Beijing 100191, China.; ^6^ Beijing Genome Precision Technology Co. Ltd., Beijing 100176, China.; ^7^ Department of Biochemistry and Molecular Biology, School of Basic Medical Sciences, Hangzhou Normal University, Hangzhou 311121, China.; ^8^ Shandong Provincial Key Laboratory of Precision Oncology, Shandong Cancer Hospital and Institute, Shandong First Medical University and Shandong Academy of Medical Sciences, Jinan 250117, China.; ^9^ Laboratory of Molecular Genetics, Republican Specialized Scientific and Practical Medical Center of Oncology and Radiology of Uzbekistan, Tashkent 100109, Uzbekistan.; ^10^ Musculoskeletal Tumor Center, Beijing Key Laboratory for Musculoskeletal Tumors, Peking University People’s Hospital, Beijing 100037, China.; ^11^ Department of Hematology, Shanxi Bethune Hospital, Shanxi Academy of Medical Sciences, Taiyuan 030032, China.

## Abstract

Immunoglobulin heavy chain (*IGH*) enhancer translocations drive multiple myeloma (MM), but routine diagnostics resolve only 32 to 38% of cases. Here, we introduce TransFinder, a multiomic pipeline integrating SMRT long-read sequencing (LRS), Hi-C, H3K27ac ChIP-seq/CUT&Tag, and RNA-seq to systematically resolve cryptic translocations and their hijacked oncogenes by deconvoluting enhancer-promoter neo-loops at rearrangement breakpoints. Applied to MM, TransFinder identified that t(16;22)(q23;q11), a canonical translocation known to activate *MAF*, also hijacks enhancers to activate *RAB36*. It further uncovered previously unrecognized t(5;8)(q35;q24) and t(1;22)(q25;q13), which activate *MYC* and *CBX7*, respectively, through chromatin topology rewiring. CRISPR-Cas9–engineered t(1;22)(q25;q13) recapitulated *CBX7* induction, and pharmacological inhibition of CBX7 suppressed myeloma cell proliferation. Beyond MM, TransFinder identified enhancer hijacking in chronic myeloid leukemia, where t(9;22)(q34;q11) repositioned an enhancer to activate *BCR-ABL1*, and pancreatic ductal adenocarcinoma, where t(2;12)(p24;q24) hijacked an enhancer to activate *SDC1*. Linking 3D genome architecture to oncogene activation, TransFinder decodes oncogenic structural variants and their trans-activities, establishing clinically actionable dependencies of noncoding drivers in malignancies.

## INTRODUCTION

Multiple myeloma (MM), the second most prevalent hematologic malignancy after lymphoma, is characterized by clonal plasma cell proliferation in the bone marrow, leading to systemic complications such as osteolytic lesions, renal impairment, and immunodeficiency (*1*, *2*). Myeloma cells exhibit a complex genetic landscape involving copy number alterations (e.g., 1q21 amplification and 17p13 deletion) and immunoglobulin heavy chain (*IGH*) translocation, which contribute to variability in treatment response and prognosis (*1*). *IGH* translocations often juxtapose the *IGH* enhancer with oncogenes, such as t(4;14) (*NSD2/FGFR3*), t(14;16) (*MAF*), t(11;14) (*CCND1*), and t(14;20) (*MAFB*), thereby driving malignant expansion (*3*). However, only 32 to 38% of patients with MM have canonical *IGH* translocations that are detected by fluorescence in situ hybridization (FISH) (*4*–*6*), implying that many pathogenic rearrangements remain cryptic with standard assays.

These structural variations (SVs) can reconfigure the three-dimensional (3D) genome and aberrantly activate oncogenes through mechanisms such as enhancer hijacking—a process in which a distal enhancer is misplaced next to a proto-oncogene, leading to its aberrant activation (*7*–*9*). While enhancer hijacking is exemplified by classical *IGH* translocations in myeloma, many noncanonical events remain undetected due to limitations in short-read sequencing and high-throughput chromosome conformation capture (Hi-C), which fail to resolve complex breakpoints and assign sequence identities of the rearranged sequences from interacting fragments, respectively (*10*–*12*). Thus, a comprehensive pipeline that integrates SV detection with 3D regulatory context is urgently needed to uncover hidden oncogenic circuits.

Myeloma cells display widespread 3D genome reorganization, including shifts in A/B compartment status and disruption of topologically associating domains (TADs) (*13*–*17*). Meanwhile, chromatin looping is widely recognized as a means for enhancer-promoter (E-P) communication, with active enhancers playing key roles in regulatory networks that drive myelomagenesis (*18*, *19*). An *IGH* translocation can reposition a powerful enhancer normally insulated within the *IGH* locus onto a previously unconnected gene locus, creating a neo-regulatory circuit between the hijacked enhancer and the oncogene’s promoter, thereby driving pathological transcription reprogramming (*3*, *10*). Thus, genome-wide characterization of SV-associated enhancer hijacking is needed to improve understanding of myelomagenesis and inform targeted therapeutic strategies.

In the current study, we found that in a cohort of 1032 patients with MM, 65.1% of cases exhibited unidentified cytogenetic abnormalities. We introduced TransFinder, a multiomic pipeline integrating single-molecule real-time (SMRT) long-read sequencing (LRS)–based SV detection, copy number variation (CNV)–normalized Hi-C reconstruction, and epigenomic profiling [H3K27ac ChIP-seq/CUT&Tag and RNA sequencing (RNA-seq)] to identify translocation-associated enhancer hijacking events that drive aberrant oncogenic transcription. Using this pipeline, we found that t(16;22) coactivates *RAB36* alongside *MAF* and identified t(5;8)(q35;q24) and t(1;22)(q25;q13) as enhancer hijacking translocations that activate *MYC* and *CBX7* transcription, respectively. We validated the t(1;22)(q25;q13) translocation by FISH and through analysis of DNA sample, G-banding, and optical genome mapping (OGM) datasets. We engineered a de novo t(1;22) translocation by CRISPR-Cas9 and investigated its impact on *CBX7* activation and myeloma cell proliferation. Extending to other malignancies, TransFinder also identified translocation-induced enhancer hijacking events in chronic myeloid leukemia (CML) and pancreatic ductal adenocarcinoma (PDAC).

## RESULTS

### Cryptic cytogenetic abnormalities in MM associate with survival heterogeneity and implicate translocation-dominant 3D genome disruption

Immunoglobulin heavy chain (*IGH*) enhancer translocations are well-established drivers of myelomagenesis, yet FISH detects canonical events in only 32 to 38% of patients with MM (*4*–*6*), leaving many with unresolved cytogenetic abnormalities. We retrospectively analyzed 1050 patients with MM assessed at two Beijing centers over 6 years. Among 1032 patients tested by FISH (table S1), canonical *IGH* translocations—t(4;14)(p16;q32) (*NSD2*/*FGFR3*), t(14;16)(q32;q23) (*MAF*), and t(11;14)(q13;q32) (*CCND1*)—were detected in 34.9% (360 of 1032), consistent with prior reports (*4*–*6*). Specifically, t(4;14) occurred in 14.4%, t(14;16) in 1.6%, and t(11;14) in 18.8%. Notably, 65.1% (672 of 1032) showed unidentified cytogenetic abnormalities, defined as *IGH* rearrangements not involving known canonical partner loci or cases without detectable *IGH* split signals, including t(14;undefined) in 25.7% (265 of 1032) and no *IGH* split in 39.4% (407 of 1032) (Fig. 1A).

**Fig. 1.**
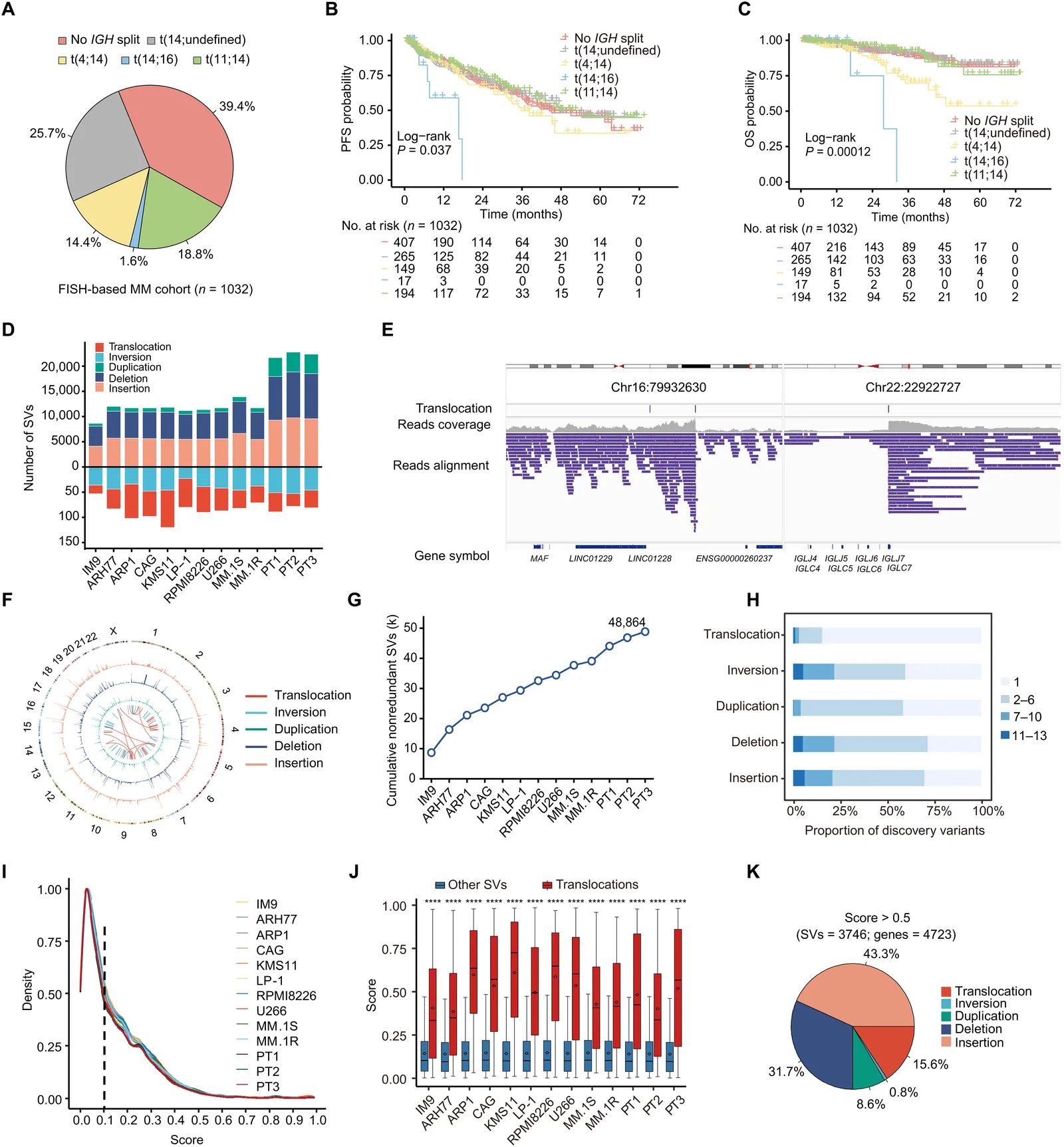
Translocation-mediated 3D chromatin remodeling underlies pathogenic structural variants in myeloma. (**A**) The proportion of canonical *IGH* translocations and unresolved cytogenetic abnormalities in a multicenter MM cohort (*n* = 1032). (**B** and **C**) Kaplan-Meier curves for PFS (B) and OS (C) by *IGH* rearrangement status: no *IGH* split (*n* = 407), t(14;undefined) (*n* = 265), t(4;14) (*n* = 149), t(14;16) (*n* = 17), and t(11;14) (*n* = 194). Plus signs indicate censoring; numbers at risk are shown below. Survival distributions among the five groups were compared using overall log-rank tests. (**D**) Per-sample structural variant spectrum displayed as a split-scale stacked bar plot. High-frequency SV classes are shown above the baseline, and rare SV classes are shown below the baseline on an expanded scale. Colors indicate SV classes. (**E**) An IGV image showing the t(16;22)(q23;q11) translocation involving the *IGL* and *MAF* in RPMI8226 cells. (**F**) A Circos plot illustrating SVs identified in the PT1 through pbsv analysis. Tracks from the outermost to innermost circles represent insertions, deletions, duplications, inversions, and translocations. (**G**) Cumulative nonredundant SV discovery curve generated by sequentially merging and deduplicating SV calls across samples. (**H**) Frequency distribution of each SV class among all myeloma samples. (**I**) A density plot showing the distribution of predictive scores for each SV from myeloma samples as estimated by the PhenoSV algorithm. (**J**) Box plots comparing PhenoSV prediction scores between translocations and other SVs across 13 myeloma samples. Statistical significance was assessed within each sample using two-sided Wilcoxon rank sum tests. *P* values were adjusted across the 13 comparisons using the Benjamini-Hochberg method. ****P < 0.0001. (**K**) The proportion of each SV type with a predictive score above 0.5 for the myeloma samples.

We next examined clinical outcomes across cytogenetic subgroups. Median progression-free survival (PFS) was longest in t(11;14) (46.3 months) and shortest in t(14;16) (16.6 months), consistent with prior work (*4*). Patients with t(14;undefined) and no *IGH* split had comparable PFS (47.5 and 44.4 months, respectively), similar to the t(11;14) subgroup. Overall survival (OS) likewise was notably longer in these three groups than in t(14;16) and t(4;14) patients (Fig. 1, B and C), suggesting clinically meaningful lesions beyond canonical *IGH* calls.

To define the genomic basis of these cryptic lesions, we obtained bone marrow samples from 21 patients with MM and analyzed 10 myeloma cell lines. CD138^+^ plasma cells were enriched by immunomagnetic selection, and three samples (PT1 to PT3) yielded >5 × 10^6^ viable cells for deep multiomic profiling (fig. S1 and table S2). We performed SMRT LRS (*20*) for base pair resolution SV mapping and profiled 3D genome organization by in situ Hi-C (cell lines) and Micro-C XL (*21*) (patients), together with H3K27ac ChIP-seq/CUT&Tag, ATAC-seq, and RNA-seq to link regulatory state, chromatin topology, and transcriptional output (fig. S1).

SMRT-seq achieved a median read length of 12,729 bp (fig. S2A). The generated libraries yielded approximately 16 to 71 Gb of mapped sequence data, corresponding to an average genome coverage of approximately 5× to 23× across samples (fig. S2B). Across pooled SV calls, insertions (45.7%) and deletions (42.9%) accounted for the largest fractions of total SVs, followed by duplications (10.7%), inversions (0.3%), and translocations (0.3%) (Fig. 1D). IGV inspection confirmed known events, including t(16;22)(q23;q11) involving *IGL* and *MAF* in RPMI8226 cells (Fig. 1E). Most SVs were short (≤400 bp) (fig. S2C) and enriched near chromosome termini (Fig. 1F and fig. S3, A and B) and transcription start sites (TSSs) (fig. S3C). We next sequentially merged SV calls across samples while removing redundant events, generating a catalog that reached 48,864 after all samples were included (Fig. 1G and table S3). Stratification by sample recurrence showed that translocations were the most sample-specific SV class, with ∼85% detected in only one sample (Fig. 1H). Using PhenoSV (*22*), translocations showed higher impact scores than other SV types (Fig. 1, I and J); among 3746 high-impact SVs (PhenoSV > 0.5), translocations comprised 15.6% despite representing only 0.3% of all SVs (Fig. 1K), indicating their disproportionate role in gene dysregulation.

To link SVs with transcription, we classified genes by SV proximity. Genes were considered associated with insertions, deletions, or duplications when these events occurred within gene bodies, whereas genes within 2 Mb of inversion or translocation breakpoints were considered associated with the respective SVs, reflecting genomic association rather than direct functional impact. Approximately 30% of genes fell within SV-associated regions, including ∼5% near translocation breakpoints (fig. S4A). Although proximity does not establish causality, these groups showed distinct expression trends. While most SV types correlated with reduced expression, genes near translocation breakpoints were expressed at higher levels (fig. S4B), consistent with enhancer rewiring–driven oncogene activation in myelomagenesis.

Next, we assessed the impact of SVs on chromatin topology. Hi-C and Micro-C XL quality control indicated robust intrasample consistency and intersample reproducibility (fig. S5A). Insulation score analysis using cworld (*15*) showed that breakpoints of inversions and translocations had notably reduced insulation relative to flanking regions (fig. S5B), resembling TAD boundary insulation. These findings indicate that translocations, although rare, exert outsized effects on 3D genome architecture and transcriptional regulation in MM.

### TransFinder integrates LRS and 3D interactomes to prioritize clinically actionable translocations

To systematically define functional chromosomal translocations in myeloma, we developed TransFinder (Translocation Finder), an integrative pipeline combining SMRT-seq, Hi-C, ChIP-seq/CUT&Tag, and RNA-seq (Fig. 2A). TransFinder first calls translocations independently of SMRT-seq and Hi-C and then nominates high-confidence events supported by both datasets. SMRT-seq–resolved base pair breakpoints and translocation orientations, together with CNV-normalized Hi-C contact maps, are used to locally reconstruct 3D chromatin architecture around each breakpoint and infer interchromosomal neo-loops. Last, TransFinder integrates epigenomic and transcriptomic annotations to connect neo-loops with regulatory output, prioritizing E-P neo-loops by mapping active enhancers and highly expressed genes [transcripts per million (TPM) > 10] and assigning their candidate oncogenic targets.

**Fig. 2.**
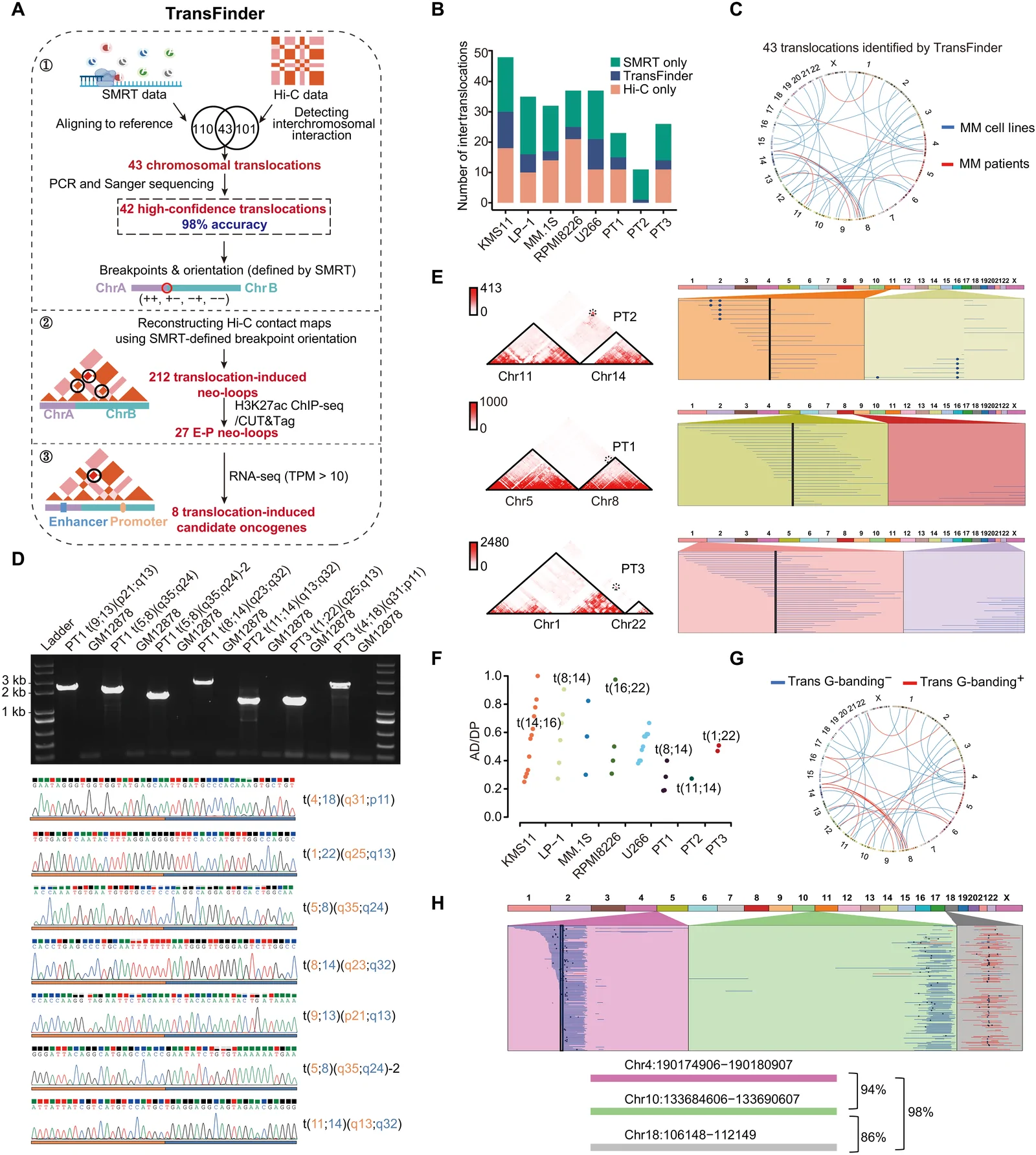
TransFinder identifies high-confidence clinically actionable translocations by integrating LRS and 3D contact maps. (**A**) Schematic illustration of TransFinder, an analytical pipeline designed to detect interchromosomal translocations and enhancer hijacking events by integrating SMRT-seq, Hi-C, ChIP-seq/CUT&Tag for H3K27ac, and RNA-seq data. (**B**) A bar plot showing the number of interchromosomal translocations across eight myeloma samples as detected from SMRT-seq alone, Hi-C alone, and both SMRT-seq and Hi-C data using TransFinder. (**C**) Circos plot showing 43 interchromosomal translocations identified by TransFinder across myeloma cell lines and patient-derived samples. (**D**) Validation of the high-confidence translocations through PCR and Sanger sequencing in patient-derived myeloma samples. (**E**) Representative examples of high-confidence translocations observed in patient-derived samples: t(11;14)(q13;q32) in PT2, t(5;8)(q35;q24) in PT1, and t(1;22)(q25;q13) in PT3. (**F**) Estimation of the prevalence of experimentally confirmed high-confidence translocations in myeloma samples using allelic depth (AD) and depth of coverage (DP) metrics derived from SMRT-seq data. (**G**) Circos plot showing experimentally confirmed high-confidence translocations, also supported by G-banding datasets. Blue lines represent translocations without G-banding support (trans G-banding^−^), while red lines represent translocations with G-banding support (trans G-banding^+^). (**H**) A complex case of translocation t(4;10;18) in LP-1 cells and GM12878 cells, with a comparative sequence homology analysis of 3-kb regions flanking each breakpoint across the involved chromosomes.

We applied TransFinder to eight myeloma samples and identified interchromosomal translocations at high confidence. SMRT-seq and Hi-C independently detected 153 and 144 events, respectively, with 43 supported by both datasets, including 35 in myeloma cell lines and 8 in patient-derived samples (Fig. 2, B and C). Of these, 42 were validated by junction-spanning polymerase chain reaction (PCR) followed by Sanger sequencing, yielding a 98% validation rate (Fig. 2D, fig. S6, and table S4). Among the validated events, three corresponded to previously reported translocations [t(16;22)(q23;q11), t(8;14)(q24;q32), and t(11;14)(q13;q32)], while the remaining events represented noncanonical translocations. Validated events included the canonical t(11;14)(q13;q32) in PT2, as well as two previously unrecognized translocations t(5;8)(q35;q24) in PT1 and t(1;22)(q25;q13) in PT3 (Fig. 2E).

To infer clonal dominance, we quantified allelic depth (AD) and depth of coverage (DP) from SMRT-seq for the 42 validated translocations. Canonical events including t(14;16)(q32;q23), t(8;14)(q24;q32), and t(16;22)(q23;q11) exhibited the highest AD/DP values in KMS11, LP-1, and RPMI8226 cells, respectively. Likewise, t(8;14)(q23;q32) in PT1, t(11;14)(q13;q32) in PT2, and t(1;22)(q25;q13) in PT3 ranked highest by AD/DP (Fig. 2F), consistent with these translocations marking dominant clones.

To evaluate prevalence, we interrogated a G-banding dataset of 1463 MM cases. Thirteen TransFinder-identified translocations were supported by G-banding (Fig. 2G and table S5), whereas submicroscopic SVs and their associated oncogenic rewiring were not captured by this approach (*23*). We also noted a complex t(4;10;18) called by SMRT-seq in both LP-1 and the normal lymphoblastoid line GM12878 but not classified by TransFinder as a bona fide translocation. Sequence comparison of ±3-kb regions flanking the three putative breakpoints revealed >85% identity (Fig. 2H), suggesting local homology as a likely source of SMRT-seq–only false positives. Collectively, these results reveal that TransFinder enables accurate, high-confidence identification of functionally relevant translocations while reducing false discovery from single-modality calls.

### Translocation-induced enhancer hijacking drives context-specific oncogene activation in myeloma

Translocation-driven oncogene activation depends on both enhancer activity and the frequency of E-P contacts (*7*). Using TransFinder, we mapped enhancer hijacking events associated with high-confidence translocations in myeloma and classified them as canonical or previously unrecognized translocations involving known or unknown oncogenic targets (Fig. 3A and table S6). We first examined canonical enhancer hijacking events linked to established myeloma drivers (*2*). In PT2, t(11;14)(q13;q32) hijacked *IGH* enhancer to the *CCND1* locus, consistent with an accessible *CCND1* promoter and high *CCND1* mRNA abundance (Fig. 3B). In LP-1 cells, t(8;14)(q24;q32) juxtaposed an enhancer from chromosome 14 to *MYC*, which was confirmed by circular chromosome conformation capture (4C-seq) anchored at the *MYC* promoter (Fig. 3C). In RPMI8226, t(16;22)(q23;q11) similarly redirected an enhancer from chromosome 22 to activate *MAF* transcription (Fig. 3D).

**Fig. 3.**
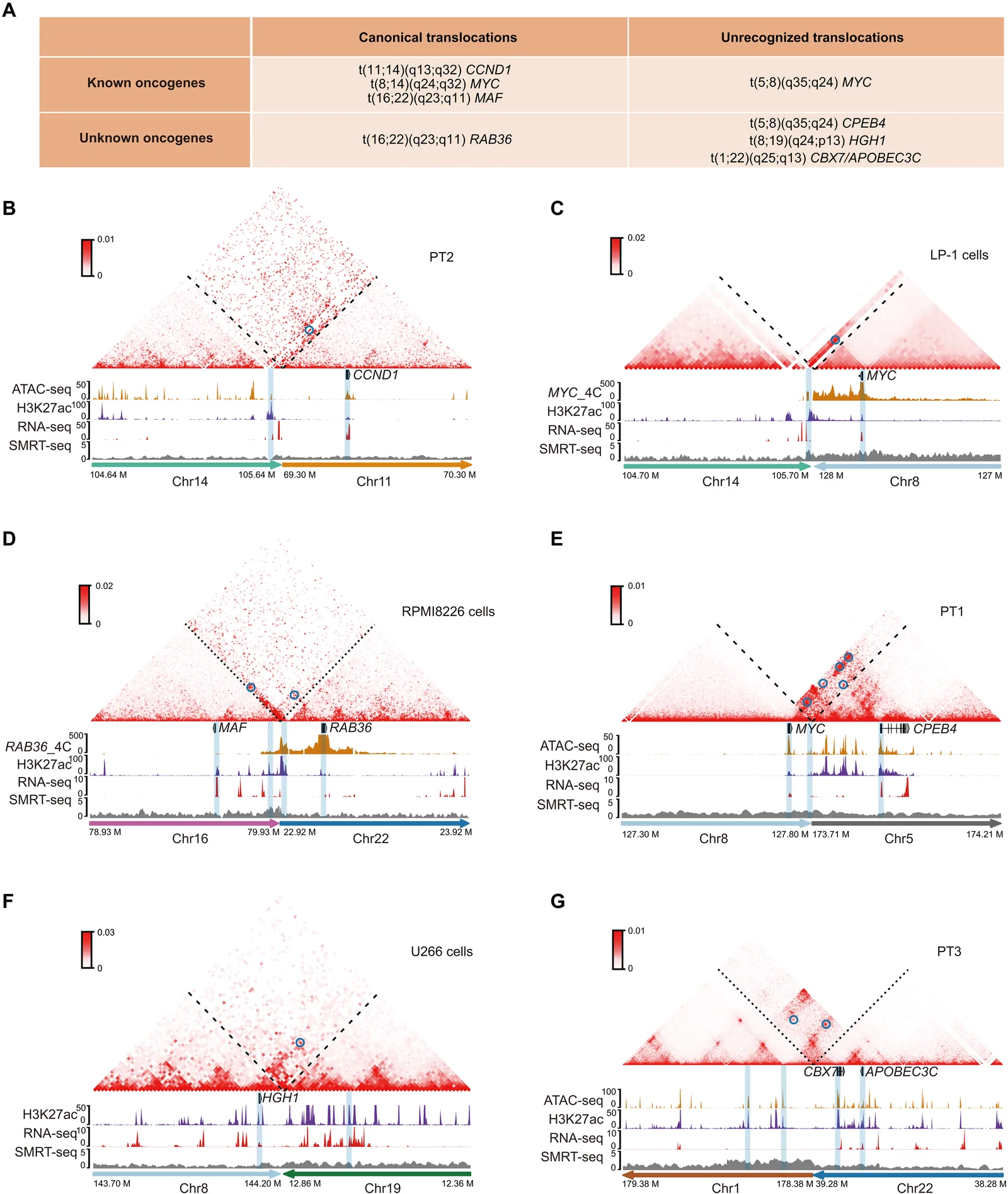
TransFinder uncovers translocation-driven enhancer hijacking underlying oncogenic transcriptional circuitry in myeloma. (**A**) Identification of enhancer hijacking events induced by translocation and implicated oncogenes in myeloma samples using TransFinder. (**B** to **G**) Sequential illustrations from top to bottom integrate Hi-C/Micro-C, ATAC-seq, 4C-seq (targeting *MYC* and *RAB36* promoters), ChIP-seq/CUT&Tag for H3K27ac, RNA-seq, and SMRT-seq data, elucidating t(11;14)(q13;q32) (*IGH/CCND1*) in the PT2 sample, t(8;14)(q24;q32) (*MYC*) in LP-1 cells, t(16;22)(q23;q11) (*MAF/RAB36*) in RPMI8226 cells, t(5;8)(q35;q24) (*MYC/CPEB4*) in the PT1 sample, t(8;19)(q24;p13) (*HGH1*) in U266 cells, and t(1;22)(q25;q13) (*CBX7/APOBEC3C*) in the PT3 sample. E-P neo-loops induced by these translocations are highlighted with blue circles, with enhancers and promoters represented by light blue bars.

A single translocation can also create multiple enhancer hijacking circuits (*24*). Consistent with this, in RPMI8226 cells, we found that t(16;22)(q23;q11) not only activates *MAF* (*2*) but also hijacks an additional enhancer from chromosome 16 to the *RAB36* locus (Fig. 3D). This interaction was validated by 4C-seq anchored at the *RAB36* promoter, supporting translocation-induced E-P looping and coordinated expression of *RAB36* with *MAF* (Fig. 3D). These data indicate that canonical translocations can simultaneously rewire multiple regulatory programs and nominate *RAB36* as a potential therapeutic vulnerability in MM harboring t(16;22)(q23;q11).

Oncogene activation can be driven by distinct translocations, as exemplified in MM where *MAF* is activated by either t(16;22)(q23;q11) or t(14;16)(q32;q23) (*3*). To extend this concept, we next focused on previously unrecognized translocations that induce enhancer hijacking and activate known oncogenes. In contrast to the canonical t(8;14)(q24;q32), which activates *MYC* transcription validated by 4C-seq, we identified an unrecognized t(5;8)(q35;q24) in the PT1 sample. This rearrangement juxtaposed a cluster of enhancers on chromosome 5 to *MYC*, accompanied by an accessible *MYC* promoter and high *MYC* mRNA abundance (Fig. 3E). Notably, the same t(5;8) event also linked an enhancer on chromosome 8 to *CPEB4*, again coinciding with promoter accessibility and high *CPEB4* expression (Fig. 3E). Additional unrecognized events included t(8;19)(q24;p13) in U266 cells, which connected an enhancer on chromosome 19 to *HGH1* (Fig. 3F), and t(1;22)(q25;q13) in PT3, which linked hijacked enhancers on chromosome 1 to the promoters of *CBX7* and *APOBEC3C*, with concordant promoter accessibility and increased transcript levels (Fig. 3G). To contextualize these expression changes, we compared the nominated target genes in translocation-positive samples with HD1, a normal plasma cell RNA-seq control from healthy donor bone marrow, and found that the corresponding targets were expressed at higher levels than in HD1 (fig. S7). Collectively, these results demonstrate that TransFinder enables high-resolution identification of diverse enhancer hijacking configurations spanning canonical and previously unrecognized translocations that activate both established and previously unappreciated oncogenes in myeloma.

### Orientation-aware reconstruction improves detection of functional translocation neo-loops

Although Hi-C–based frameworks such as EagleC (*25*) can sensitively detect interchromosomal translocations from contact maps, inferring translocation orientation from Hi-C alone remains difficult, particularly in complex cancer genomes where interaction patterns can be ambiguous. To systematically evaluate alternative orientation-calling strategies, we compared SMRT-seq– and Hi-C–assigned orientations for high-confidence translocations across eight myeloma samples. Among the 42 TransFinder-identified translocations, 36 (85.7%) showed concordant orientations, whereas 6 (14.3%) were discordant (Fig. 4A). Orientation mismatches occurred for t(1;7)(p12;q31) and t(8;14)(q24;q32) in LP-1, t(8;13)(q21;q12) in MM.1S, t(2;9)(q34;q33) in RPMI8226, t(8;14)(q23;q32) in PT1, and t(11;14)(q13;q32) in PT2 (Fig. 4B), revealing an intrinsic limitation of orientation inference based solely on Hi-C contact patterns. To understand the source of these discrepancies, we inspected Hi-C contact maps around the six discordant events. In each case, the breakpoint positions inferred from Hi-C were close to but did not precisely match the SMRT-seq–defined breakpoints (Fig. 4C). Because Hi-C identifies translocations indirectly through interaction signatures rather than breakpoint-spanning reads, modest positional uncertainty can translate into incorrect or ambiguous orientation calls.

**Fig. 4.**
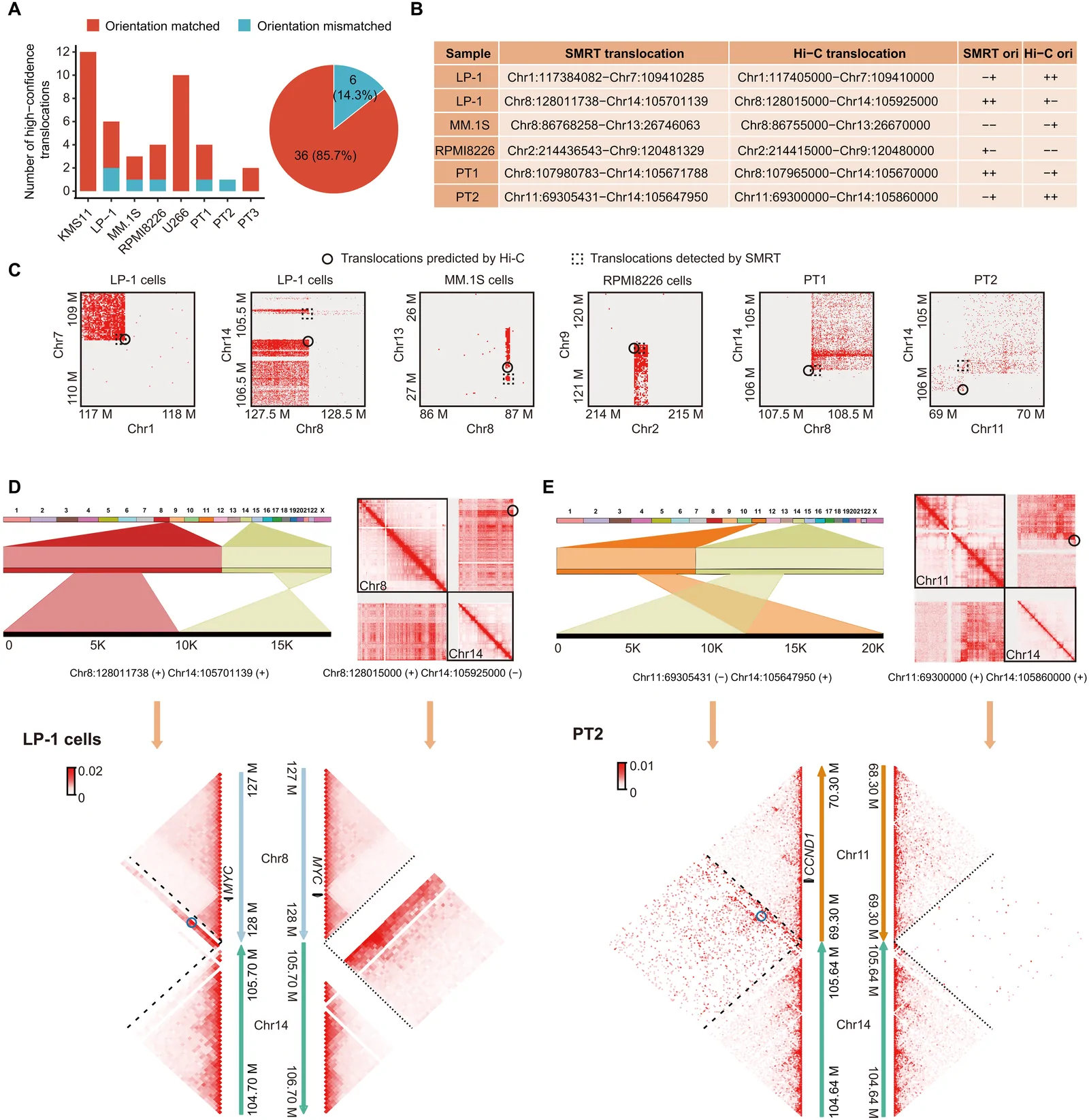
Accurate determination of translocation orientation is essential for reconstructing rearranged chromatin architecture. (**A**) Distribution of high-confidence chromosomal translocations identified across eight myeloma samples, classified by concordant or discordant breakpoint orientation between Hi-C and SMRT-seq data. (**B**) Summary of six translocations exhibiting discordant breakpoint orientation between Hi-C and SMRT-seq, including genomic coordinates and inferred orientations. SMRT ori, SMRT-orientation; Hi-C ori, Hi-C orientation. (**C**) Hi-C contact maps of the six translocations with discordant orientation assignments. Circles indicate breakpoint positions inferred from Hi-C contact patterns, and dashed boxes denote breakpoint positions defined by SMRT-seq. (**D**) Reconstruction of rearranged chromatin architecture for the t(8;14)(q24;q32) translocation in LP-1 cells. Chromatin structure was reconstructed on the basis of breakpoint orientation defined by SMRT-seq (top) or inferred from Hi-C (bottom). (**E**) Reconstruction of rearranged chromatin architecture for the t(11;14)(q13;q32) translocation in the PT2 sample. Chromatin structure was reconstructed using breakpoint orientation defined by SMRT-seq (left) or inferred from Hi-C (right). The contact maps in (E) and (D) are also shown in Fig. 3 (B and C), respectively, and are included here to compare the effect of breakpoint orientation on chromatin reconstruction.

We next tested the functional consequences of orientation choice on chromatin architecture reconstruction, focusing on two pathogenic myeloma translocations t(8;14)(q24;q32) and t(11;14)(q13;q32), which activate *MYC* and *CCND1*, respectively (Fig. 4, D and E). Reconstructions based on Hi-C–derived translocation orientations failed to recover the expected neo-loops at the junctions, limiting identification of the regulatory connections between distal enhancers and *MYC* or *CCND1*. In contrast, using SMRT-seq–defined orientations faithfully restored the characteristic junction neo-loops at both *loci* and revealed E-P contacts converging on *MYC* in LP-1 and *CCND1* in PT2. Collectively, these results demonstrate that accurate orientation assignment is essential for faithful reconstruction of rearranged chromatin architecture. By prioritizing LRS-defined breakpoint orientation for translocations supported by both LRS and Hi-C, TransFinder enables robust identification of translocation-induced neo-loops and their associated candidate oncogenes.

### Recurrent t(1;22)(q25;q13) hijacks a distal enhancer to activate *CBX7* and myeloma cell proliferation via E-P neo-looping

Given the established roles of CBX7 in hematologic malignancies, including leukemia initiation from hematopoietic stem cells (*26*) and progression of T cell and aggressive B cell lymphomas (*27*), we reasoned that t(1;22)(q25;q13) identified in PT3 and associated with distal enhancer engagement and *CBX7* activation may contribute to myeloma proliferation. To validate this event in primary myeloma cells, we developed Tn5 transposase–based FISH (Tn5-FISH) (*28*) probes targeting nonrepetitive sequences flanking the breakpoints on chromosomes 1 and 22 (table S7). This high-resolution imaging approach leverages Tn5-mediated fluorescent labeling to improve hybridization efficiency and enable subkilobase colocalization with single-molecule sensitivity for detecting complex structural variants (*28*). t(1;22) signals were detected exclusively in CD138^+^ myeloma cells from PT3, but not in matched CD138^−^ mononuclear cells or GM12878 controls, supporting a tumor-specific association of t(1;22)(q25;q13) with myeloma (Fig. 5A).

**Fig. 5.**
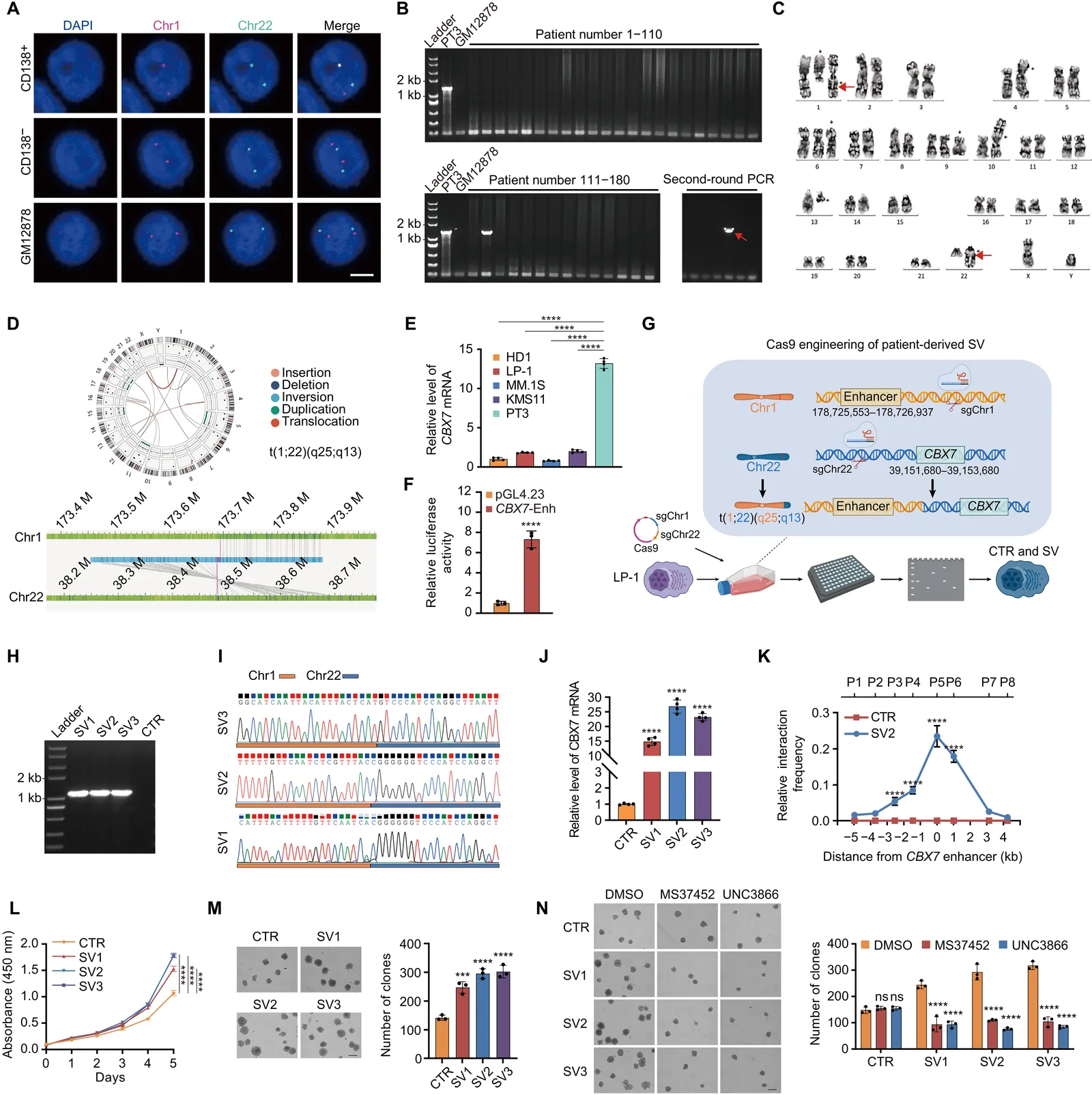
Recurrent t(1;22)(q25;q13) activates *CBX7* through enhancer hijacking in MM. (**A**) FISH validation of t(1;22)(q25;q13) in PT3. CD138^+^ and CD138^−^ indicate myeloma cells and other mononuclear cells, respectively. Scale bar, 5 μm. (**B**) PCR screening of 180 MM samples pooled in groups of five, followed by individual testing of samples from the positive pool. (**C**) G-banding showing t(1;22)(q21;q11) and (**D**) OGM showing t(1;22)(q25;q13) in separate myeloma cases. (**E**) *CBX7* mRNA levels in PT3, HD1, and t(1;22)-negative myeloma cell lines (*n* = 4). (**F**) Activity of the *CBX7*-associated hijacked enhancer measured by dual-luciferase reporter assay in LP-1 cells (*n* = 3). (**G**) CRISPR strategy for generating t(1;22)(q25;q13)-edited LP-1 clones at the PT3 breakpoints. CTR denotes the nontargeting single guide RNA (sgRNA) control clone; SV1 to SV3 denote edited clones. sgChr1 and sgChr22 target the breakpoint regions on chromosomes 1 and 22, respectively. Created in BioRender. Li, Q. (2026) https://BioRender.com/lx8ndpn. (**H**) Junction-spanning PCR and (**I**) Sanger sequencing confirming t(1;22)(q25;q13) in SV1 to SV3. (**J**) *CBX7* mRNA levels in SV1 to SV3 relative to CTR (*n* = 4). (**K**) 3C-qPCR analysis of *CBX7* E-P contacts in SV2 relative to CTR, normalized to *GAPDH* (*n* = 4). (**L**) CCK-8 proliferation assays in CTR and SV1 to SV3 (*n* = 4). (**M**) Colony formation by CTR and SV1 to SV3 after 12 days in methylcellulose (*n* = 3). (**N**) Colony formation by CTR and SV1 to SV3 after treatment with dimethyl sulfoxide (DMSO), 10 μM MS37452, or 10 μM UNC3866 (*n* = 3). Scale bars, 200 μm (M and N). Data are mean ± SD. *P* values were calculated using one-way analysis of variance (ANOVA) with Dunnett’s test versus PT3 (E) or CTR (J and M), two-tailed unpaired Student’s *t* test (F), or two-way ANOVA (K, L, and N). ***P < 0.001 and ****P < 0.0001. DAPI, 4′,6-diamidino-2-phenylindole; ns, not significant.

To assess prevalence, we performed junction-spanning PCR on genomic DNA from 180 patients with MM using primers designed against the PT3 breakpoints. To optimize the screening efficiency, we pooled five samples per lane, with PT3 serving as a positive control. Initial screening identified one positive pool (Fig. 5B). Each sample from the positive pool was then individually analyzed by PCR and Sanger sequencing, revealing the presence of t(1;22)(q25;q13) in one patient (Fig. 5B and fig. S8A). Notably, the translocation was detected in bone marrow but not in matched blood plasma or buccal swabs, consistent with a myeloma-restricted origin (fig. S8A). We further identified one case in a G-banding cohort (*n* = 1463; Fig. 5C) and one case by OGM (*n* = 25; Fig. 5D), supporting t(1;22)(q25;q13) as a recurrent MM-associated translocation.

We next evaluated the regulatory consequence of the hijacked enhancer on *CBX7* transcription. Real-time reverse transcription quantitative PCR (qPCR) showed that *CBX7* mRNA was significantly elevated in PT3 compared with HD1 and t(1;22)(q25;q13)-negative myeloma cell lines, including LP-1, MM.1S, and KMS11 (Fig. 5E). Consistently, luciferase reporter assays in LP-1 cells transfected with pGL4.23 constructs harboring the t(1;22)(q25;q13)-hijacked enhancer showed elevated luciferase activity (Fig. 5F), supporting enhancer activity within the translocated element.

To directly test whether t(1;22)(q25;q13) is sufficient to activate *CBX7* and promote myeloma proliferation, we engineered de novo t(1;22) in LP-1 cells using CRISPR-Cas9. LP-1 cells were selected because they lacked preexisting translocations near the PT3 breakpoints and harbored the corresponding chromosome 1 enhancer (fig. S8B). Paired single guide RNAs (sgRNAs) (*29*) targeting the PT3-associated breakpoints were used to generate t(1;22)(q25;q13) (Fig. 5G), while a single LP-1 clone transduced with nontargeting sgRNAs and subjected to the same single-cell cloning workflow was used as the control. Three independently derived clones carrying t(1;22), designated SV1, SV2, and SV3, were confirmed by junction-spanning PCR and Sanger sequencing (Fig. 5, H and I). All three edited clones exhibited increased *CBX7* mRNA compared with the CTR clone; SV2 showed the strongest induction and was selected for chromatin interaction analysis (Fig. 5J).

We then quantified translocation-induced chromatin rewiring by chromosome conformation capture followed by qPCR (3C-qPCR) (*30*). Using a *CBX7* promoter anchor primer (P0) and eight interval-spaced test primers (P1 to P8), including a primer targeting the center of the hijacked enhancer (P5), we observed a marked increase in contact frequency between the *CBX7* promoter and the hijacked enhancer in SV2 relative to the CTR clone (Fig. 5K). Interaction strength decayed progressively away from P5, consistent with a focal E-P neo-loop architecture.

Functionally, all three t(1;22)-engineered clones showed significantly increased proliferation in CCK-8 assays (Fig. 5L) and enhanced colony formation (Fig. 5M) relative to the CTR clone. Pharmacologic inhibition of CBX7 with MS37452 (*31*) or UNC3866 (*32*), chromodomain-targeting compounds that block CBX7 recognition of H3K27me3, significantly reduced both proliferation (fig. S8C) and colony formation (Fig. 5N) in all three t(1;22)-edited clones, whereas CTR cells were largely unaffected. This selective sensitivity supports a CBX7-dependent growth advantage conferred by the translocation. Collectively, these results support a model in which recurrent t(1;22)(q25;q13) hijacks a distal enhancer to activate *CBX7* via E-P neo-looping, thereby promoting myeloma proliferation and revealing CBX7 as a potential therapeutic vulnerability in MM.

### TransFinder reveals conserved enhancer hijacking principles across hematologic and epithelial malignancies

To evaluate the broader applicability of TransFinder, we analyzed translocation-induced enhancer hijacking events in additional cancer types: CML and PDAC. We assembled publicly available multiomic datasets including SMRT-seq, Hi-C, H3K27ac ChIP-seq, and RNA-seq, from the CML cell line K562 and PDAC cell lines BxPC3 and PANC1. TransFinder identified 30 high-confidence translocations in BxPC3, 8 in PANC1, and 5 in K562 (Fig. 6A), along with 10 associated oncogenes: 6 in BxPC3 (e.g., *SDC1* and *FOXC1*), 3 in PANC1 (e.g., *CNDP2*), and *BCR-ABL1* in K562 (Fig. 6B).

**Fig. 6.**
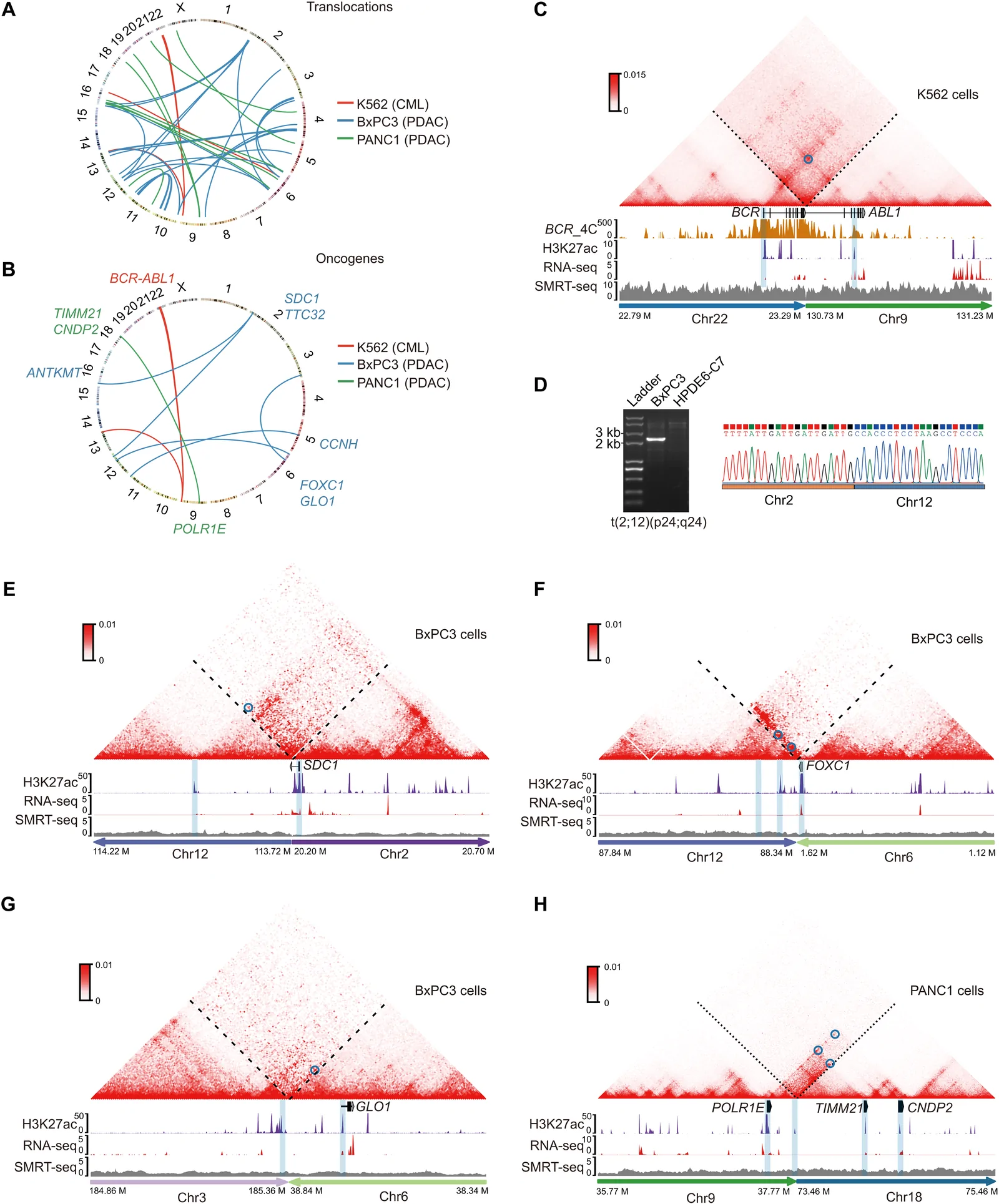
TransFinder uncovers translocation-induced enhancer hijacking in CML and PDAC. (**A**) Identification of chromosomal translocations in K562, BxPC3, and PANC1 cell lines using TransFinder. (**B**) Identification of translocation-induced oncogenes in K562, BxPC3, and PANC1 cell lines using TransFinder. (**C**) Sequential illustrations from top to bottom integrate Hi-C, 4C-seq targeting the *BCR* promoter, ChIP-seq for H3K27ac, RNA-seq, and SMRT-seq data, elucidating t(9;22)(q34;q11) (*BCR-ABL1)* in K562 cells. E-P neo-loops induced by these translocations are highlighted with blue circles, with enhancers and promoters represented by light blue bars. (**D**) PCR and Sanger sequencing of t(2;12)(p24;q24) in BxPC3 cells, with HPDE6-C7 cells as a control. (**E** to **H**) Sequential illustrations from top to bottom integrate Hi-C, ChIP-seq for H3K27ac, RNA-seq, and SMRT-seq data, elucidating t(2;12)(p24;q24) (*SDC1*), t(6;12)(p25;q21)(*FOXC1*), and t(3;6)(q27;p21)(*GLO1*) in BxPC3 cells; t(9;18)(p13;q22)(*CNDP2*, *TIMM21*, *POLR1E*) in PANC1 cells. E-P neo-loops induced by these translocations are highlighted with blue circles, with enhancers and promoters represented by light blue bars.

In K562 cells, TransFinder detected the established t(9;22)(q34;q11) translocation that generates *BCR-ABL1*, which encodes a chimeric protein with constitutive kinase activity to drive CML pathogenesis (*33*). The same rearrangement also linked a chromosome 9 enhancer to *BCR-ABL1* locus, accompanied by high *BCR-ABL1* expression (Fig. 6C). This event was validated by 4C-seq targeting the *BCR* promoter (Fig. 6C), suggesting that *BCR-ABL1* transcription activation is driven by the formation of an E-P neo-loop induced by t(9;22)(q34;q11). Thus, a single translocation could simultaneously create a fusion oncogene and reinforce its transcription through chromatin reorganization.

In PDAC, TransFinder identified a previously unrecognized translocation t(2;12)(p24;q24) in BxPC3 cells, which was validated by junction-spanning PCR and Sanger sequencing (Fig. 6D). This translocation linked a chromosome 12 enhancer to the *SDC1* locus (*34*) (encoding a transmembrane organizer implicated in tumor invasion and angiogenesis) and was accompanied by high *SDC1* expression (Fig. 6E). TransFinder identified additional translocations and their implicated oncogenes in PDAC, including t(6;12)(p25;q21) that affects *FOXC1* (*35*) (encoding a transcription factor governing cellular differentiation), t(3;6)(q27;p21) that targets *GLO1* (*36*) (encoding a glyoxalase regulating oxidative stress response), and t(9;18)(p13;q22) that simultaneously affects *CNDP2* (*37*) (encoding an aminopeptidase), *TIMM21* (*38*) (encoding an inner mitochondrial membrane translocase), and *POLR1E* (*39*) (encoding an RNA polymerase I subunit) (Fig. 6, F to H).

To further assess the functional relevance of TransFinder-nominated genes, we integrated DepMap Chronos CRISPR gene effect scores with matched RNA-seq expression data (*40*). *MYC*, *CCNH*, *CCND1*, and *POLR1E* were classified as common essential genes in DepMap models (fig. S9A). We next examined the association between target gene expression and CRISPR dependency across DepMap cell lines. Ten of the 18 nominated genes showed negative Spearman correlations between expression and Chronos gene effect scores, indicating that higher expression was associated with stronger dependency for a subset of targets (fig. S9B). These heterogeneous dependency patterns suggest that some TransFinder-nominated genes represent broadly required cancer dependencies, whereas others may confer expression-associated vulnerabilities in specific regulatory or structural-variant contexts. Collectively, these findings demonstrate that TransFinder identifies diverse translocation-driven enhancer hijacking events across hematologic and solid malignancies, supporting its broader applicability for resolving oncogenic rewiring through chromatin topology alterations.

## DISCUSSION

A large fraction of MM remains cytogenetically unassigned by routine clinical assays despite marked biological and clinical heterogeneity. We propose that this “unexplained” space is not merely technical noise but is enriched for a major class of noncoding regulatory lesions, including translocation-induced enhancer hijacking, where active enhancers are ectopically repositioned to drive oncogene expression through E-P neo-loops. This perspective reframes translocations in MM from static, locus-based abnormalities to 3D regulatory rewiring events, whose pathogenicity is dictated by enhancer activity, contact topology, and transcriptional output.

To operationalize this framework, we introduced TransFinder, which integrates SMRT LRS, CNV-normalized Hi-C, and orthogonal epigenomic/transcriptomic layers (H3K27ac ChIP-seq/CUT&Tag and RNA-seq) to prioritize translocations with interpretable regulatory consequences. This integrative design addresses the complementary limitations of single-modality analyses: Breakpoint calling without regulatory context can yield large numbers of variants of uncertain functional relevance, whereas contact map–based inference alone may lack sequence resolution and can misassign rearrangement geometry (*12*, *41*). By requiring concordant evidence across modalities and anchoring reconstruction on LRS-resolved breakpoints and orientation, TransFinder reduces false discovery and enables a mechanism-first interpretation, from breakpoint to neo-loop to enhancer engagement and target gene activation. Our results underscore a key methodological principle: Orientation is not optional. Accurate orientation is often difficult to infer from Hi-C patterns alone in complex cancer genomes, yet it is essential for faithful reconstruction of rearranged chromatin architecture and correct nomination of E-P neo-loops; incorporating breakpoint-spanning LRS reads provides the critical constraint that makes reconstruction testable and experimentally verifiable.

Although elevated gene expression can be readily detected by transcriptomic profiling, such analyses do not reveal the regulatory mechanisms underlying aberrant activation. By integrating SV detection with chromatin interaction data, TransFinder enables direct attribution of gene deregulation to enhancer hijacking events induced by specific chromosomal translocations, representing a regulatory mode of gene activation that cannot be inferred from gene expression data alone. The heterogeneous dependency profiles of TransFinder-nominated genes suggest that such rewiring can create either broadly shared dependencies or vulnerabilities restricted to specific expression or structural-variant contexts. This mechanistic resolution may improve interpretation of oncogenic drivers and inform therapeutic strategies targeting enhancer-dependent transcriptional programs.

More broadly, our findings emphasize that translocations do not merely generate chimeric oncogenes (e.g., *BCR-ABL1*) (*33*) but can also reprogram transcription through ectopic E-P interactions. The enhancer hijacking associated with *BCR-ABL1* in CML supports enhancer rewiring as a shared oncogenic mechanism across malignancies, consistent with pan-cancer observations that structural variants can reposition regulatory elements to ectopically activate proto-oncogenes (*7*). Together, the MM, CML, and PDAC analyses support the broader applicability of TransFinder for identifying noncoding oncogenic drivers and reconstructing the regulatory circuits imposed by SV. However, the current workflow remains constrained by its relatively high cell input requirement, and all primary samples analyzed in this study were processed from freshly isolated bone marrow mononuclear cell (BMMCs). Broader application will therefore require lower-input workflows and validation in frozen or biobanked specimens.

Emerging low-input approaches for LRS and chromatin conformation profiling, including PCR-free SMRT library preparation, low-C, and CiFi, may substantially reduce sample requirements, although their performance in primary myeloma specimens remains to be systematically validated (*42*–*44*). TransFinder is therefore best viewed as a discovery platform for deep profiling of selected high-quality primary specimens rather than as a routine clinical workflow. Once functional regulatory SVs are defined, recurrent lesions can be converted into targeted FISH or Tn5-FISH assays for larger-scale screening, whereas patient-specific junctions may support relapse or minimal residual disease tracking. This staged strategy shifts the emphasis from cataloging additional translocations to prioritizing those with functional and clinical relevance. Such lesions may refine molecular subtyping beyond canonical FISH categories and reveal vulnerabilities at either the effector gene or regulatory level.

In summary, TransFinder advances a regulome- and 3D genome-aware framework for interpreting translocations, positioning enhancer hijacking as a unifying mechanism for cryptic oncogene activation in MM and a generalizable principle across malignancies. This perspective expands the actionable landscape beyond coding mutations and fusion proteins to include noncoding regulatory architecture as a source of clinically meaningful vulnerabilities.

## MATERIALS AND METHODS

### Ethics approval and consent to participate

This study was approved by the Ethics Committee of Peking University People’s Hospital (2020LP00920). All patients and the healthy bone marrow donor provided written informed consent before any study procedure.

### Primary sample collection and plasma cell isolation

Bone marrow aspirates were collected during standard diagnostic aspiration procedures. BMMCs were isolated by density gradient centrifugation using Ficoll-Paque Plus (Cytiva; density, 1.077 g/ml) at 400*g* for 30 min at 20°C. The interphase layer containing BMMCs was harvested and washed thrice with Hanks’ balanced salt solution (Solarbio) to remove platelets and plasma contaminants. Cell pellets were obtained by centrifugation at 500*g* for 5 min at room temperature (22° to 25°C) and resuspended in 1 ml of phosphate-buffered saline (PBS; Solarbio). For erythrocyte lysis, cell suspensions were treated with 1 to 2 ml of BD Pharm lysing buffer (BD Biosciences) for 10 min at 4°C, followed by centrifugation (500*g*, 5 min, room temperature). The resulting pellets were resuspended in PBS, and viable cell counts were determined by trypan blue exclusion assay using a hemocytometer under light microscopy (viability > 95% for downstream applications). All primary bone marrow aspirates used in the TransFinder workflow were processed immediately after collection, and CD138^+^ cells were enriched from freshly isolated BMMCs.

To purify malignant plasma cells, CD138^+^ cells were isolated from BMMCs via positive immunomagnetic selection. Briefly, cells were incubated with anti-human CD138 MicroBeads (Miltenyi Biotec) for 15 min at 4°C and then loaded onto preequilibrated MS columns (Miltenyi Biotec) placed in a QuadroMACS separator. Unbound cells were washed out with PBS/0.5% bovine serum albumin (BSA), and magnetically retained CD138^+^ cells were eluted after column removal from the magnetic field. Following CD138^+^ enrichment, the approximate per-sample input requirements for the TransFinder workflow were 3 × 10^6^ CD138^+^ cells for SMRT long-read genome sequencing, 1 × 10^6^ for in situ Hi-C, 5 × 10^5^ for RNA-seq, and 5 × 10^4^ for CUT&Tag.

### Cell culture

The human MM cell lines ARP1, CAG, KMS11, LP-1, MM.1R, MM.1S, RPMI8226, U266, ARH77, and IM9 were provided by Z. Liu (Shandong Cancer Hospital and Institute). GM12878 lymphoblastoid cells were obtained from C. Li (Peking University). The CML cell line K562 was obtained from J. Lu (Peking University People’s Hospital). The PDAC cell lines BxPC3 and normal pancreatic cells HPDE6-C7 were obtained from Y. Zhao (Shanghai Institute of Biochemistry and Cell Biology, CAS). All cell lines were authenticated by short tandem repeat profiling and maintained in mycoplasma-free conditions, as confirmed by monthly testing using the Mycoplasma Detection Kit (Yeasen). Myeloma cell lines and K562 cells were cultured in RPMI 1640 medium (Corning), while BxPC3 and HPDE6-C7 cells were cultured in Dulbecco’s modified Eagle’s medium (Corning), all supplemented with 10% heat-inactivated fetal bovine serum (FBS), penicillin (100 U/ml), and streptomycin (100 μg/ml) (Macgen). RPMI8226 and U266 cells were cultured with 15% FBS for optimal growth. All cultures were maintained at 37°C in a humidified atmosphere with 5% CO_2_. The medium was refreshed every 48 to 72 hours.

### In situ Hi-C

Hi-C libraries were constructed as described (*45*) with minor modifications. Briefly, freshly isolated primary myeloma cells or cell lines (1 to 3 × 10^6^ cells per sample) were crosslinked with 1% formaldehyde (Millipore Sigma) in PBS for 10 min at 22°C with gentle agitation, followed by quenching with 0.2 M glycine. After two washes with ice-cold PBS, cells were lysed in hypotonic buffer [10 mM tris-HCl (pH 8.0), 10 mM NaCl, 0.2% NP-40, and protease inhibitors] for 15 min on ice. Nuclei were pelleted (2500*g*, 5 min, 4°C) and resuspended in NEBuffer 3.1.

Chromatin was digested with 100 U of MboI (NEB) overnight at 37°C, and restriction ends were filled with biotin-14-dATP (Invitrogen) using Klenow fragment (NEB) in 1× NEBuffer 3.1 (37°C, 4 hours). Proximity ligation was performed with T4 DNA ligase (NEB) in 1× ligation buffer containing 1% Triton X-100 (22°C, 4 hours). Crosslinks were reversed by proteinase K (20 mg/ml; TransGen Biotech) digestion at 60°C overnight.

DNA was purified by phenol:chloroform:isoamyl alcohol (25:24:1) extraction and ethanol precipitation, followed by shearing to 300- to 500-bp fragments using a Covaris S220 sonicator (50-W peak power, 20% duty factor, 200 cycles per burst, 150 s). Biotinylated DNA was captured on MyOne Streptavidin C1 beads (Invitrogen) using 2× binding buffer [10 mM tris-HCl (pH 7.5) and 2 M NaCl].

End repair and dA-tailing were performed on beads using T4 DNA polymerase/Klenow fragment (NEB), followed by adaptor ligation with Vazyme Rapid DNA Ligase and indexed adapters (15 min, 22°C). Libraries were amplified with VAHTS HiFi PCR Mix (Vazyme; 3 to 5 cycles) and size-selected (250 to 500 bp) using Hieff NGS Beads (Yeasen). Libraries were quantified and sequenced on the MGI-seq platform (BGI).

### Micro-C XL

Micro-C XL libraries were prepared following established protocols (*21*, *46*) with minor modifications. Briefly, freshly isolated bone marrow–derived myeloma cells (2 × 10^6^) were first fixed with 1% formaldehyde (Millipore Sigma) in PBS under gentle rotation at room temperature for 15 min, followed by quenching with 0.125 M glycine. Nuclei were then crosslinked with 3 mM ethylene glycol-bis (succinimidyl succinate) (Thermo Fisher Scientific) at room temperature for 40 min, terminated by 0.4 M glycine treatment and a PBS wash containing 0.05% BSA. After cell lysis, chromatin was fragmented using micrococcal nuclease (NEB) at concentrations optimized through preexperimental titration, with digestion proceeding at 37°C for 10 min before termination via 1.5 mM EGTA addition and thermal inactivation at 65°C for 10 min.

DNA end processing involved sequential 3′-dephosphorylation and 5′-phosphorylation using T4 polynucleotide kinase (NEB) at 37°C for 15 min followed by Klenow polymerase-mediated blunt-end repair incorporating biotinylated 2′-deoxycytidine 5′-triphosphate/2′-deoxyadenosine 5′-triphosphate (Invitrogen) at 25°C for 45 min. Chromatin complexes were ligated using T4 DNA ligase (NEB) at room temperature for 3 hours, after which exonuclease III (NEB) treatment selectively removed unligated biotinylated termini. Crosslink reversal was achieved through proteinase K (TransGen Biotech) digestion at 60°C for 2 hours followed by silica membrane–based DNA purification (DNeasy Blood and Tissue Kit, QIAGEN) and dual-stage size selection (250 to 500 bp) using magnetic bead fractionation (AMPure XP, Beckman Coulter).

Library preparation included blunt-end repair, polyadenylation, and adapter ligation using the VAHTS Universal Plus DNA Library Prep Kit (Vazyme), followed by streptavidin bead–based enrichment with C1 beads (Invitrogen). PCR amplification was performed under the following conditions: initial denaturation at 95°C for 3 min; 10 to 12 cycles of 98°C for 20 s, 60°C for 15 s, and 72°C for 30 s; and final extension at 72°C for 5 min. Libraries were quantified and sequenced on the MGI-seq platform (BGI).

### SMRT LRS

Genomic DNA isolated from myeloma cells via CTAB-based extraction underwent comprehensive quality assessment, including purity/concentration measurement (NanoDrop 2000, Thermo Fisher Scientific), size distribution analysis (pulsed-field gel electrophoresis), and precise quantification (Qubit Fluorometric Quantitation system, Thermo Fisher Scientific). For libraries targeting >15-kb fragments, 10.0 μg of DNA was sheared using g-TUBEs (Covaris) at 2400*g* for 2 min, followed by size selection with AMPure PB beads (Pacific Biosciences).

SMRTbell library construction (SMRTbell Express Template Prep Kit 2.0, Pacific Biosciences) involved three sequential enzymatic phases: (i) DNA damage repair using reaction mix 1 and DNA damage repair mix v2 (37°C, 30 min), (ii) end repair/A-tailing with reaction mix 2 and end prep mix (20° → 65°C thermal cycling), and (iii) adapter ligation employing overhang adapter v3 and ligation mix. Postligation purification incorporated dual-stage size exclusion using the BluePippin System (Sage Science) to eliminate sub-15-kb fragments, followed by SMRTbell library normalization in 1× elution buffer.

Primer annealing and polymerase binding were configured through SMRT Link Sample Setup using Sequencing Primer v4 (SMRTbell Express Template Prep Kit v2), with large-fragment handling facilitated by wide-bore pipette tips. Sequencing was conducted on PacBio platforms using a single 8 M SMRT Cell, with myeloma cell line libraries analyzed in continuous long read (CLR) mode on the Sequel II system and patient-derived libraries in High-Fidelity (HiFi) mode on the Revio system.

### RNA sequencing

Total RNA was extracted using the QIAGEN RNeasy Plus kit (QIAGEN), with RNA integrity verified via the RNA Nano 6000 Assay Kit on an Agilent Bioanalyzer 2100 system (Agilent Technologies). Polyadenylated mRNA was enriched from total RNA using poly-T oligo-conjugated magnetic beads and fragmented via divalent cation-mediated hydrolysis in first-strand synthesis reaction buffer under elevated temperature. First-strand cDNA synthesis was performed with random hexamer primers and M-MuLV reverse transcriptase (NEB), followed by second-strand synthesis using DNA polymerase I and ribonuclease H (RNase H) (NEB). DNA overhangs were blunt-ended via exonuclease/polymerase treatment, and 3′ ends were adenylated for Illumina-compatible adapter ligation. Size selection of cDNA fragments (370 to 420 bp) was performed using AMPure XP beads (Beckman Coulter), followed by PCR amplification using Phusion HiFi DNA polymerase and indexed primers (Vazyme). Library quality was validated on the Agilent Bioanalyzer 2100 system before paired-end sequencing (150 bp) on an Illumina NovaSeq 6000 platform.

### CUT&Tag

CUT&Tag profiling was performed using the NovoNGS CUT&Tag 4.0 High-Sensitivity Kit (Novoprotein). Briefly, 10^5^ myeloma cells isolated from patient bone marrow or cell lines were incubated with H3K27ac primary antibody (Cell Signaling Technology; 1:100 dilution) followed by goat anti-rabbit immunoglobulin G secondary antibody (Novoprotein; 1:200 dilution). Antibody-bound chromatin was complexed with protein A-Tn5 transposase, with enzymatic activation initiated through Mg^2+^ supplementation (37°C, 1 hour). Reactions were terminated using proprietary stop buffer, and DNA fragments were isolated using magnetic bead–based purification (Novoprotein). Libraries were quantified, quality-validated by fragment analysis, and sequenced in paired-end mode (150 bp) on the Illumina NovaSeq 6000 platform.

### ATAC-seq

ATAC-seq libraries were prepared using the ATAC-seq Kit (Novoprotein). Briefly, freshly isolated nuclei from 5 × 10^4^ cells were fragmented using Tn5 transposase–mediated fragmentation in tagmentation buffer (37°C, 30 min). Tagmented DNA was purified using Tagment DNA extract beads, followed by limited-cycle PCR amplification. Library quality was verified through electrophoretic assessment of nucleosomal periodicity (2% agarose gel) before paired-end sequencing (150 bp) on the Illumina NovaSeq 6000 platform.

### Breakpoint-spanning PCR and Sanger sequencing validation

Genomic DNA was extracted from 1 × 10^6^ cells using the TIANamp Genomic DNA Kit (TIANGEN). DNA purity and integrity were assessed via spectrophotometry (Thermo Fisher Scientific) and agarose gel electrophoresis. Junction-spanning primers flanking predicted translocation breakpoints were designed using National Center for Biotechnology Information (NCBI) Primer-BLAST (product size: 1 to 3 kb; genomic specificity parameters optimized). PCR amplification was performed with 2× UltraTaq PCR StarMix (GenStar), with GM12878 genomic DNA as a negative control and Sanger sequencing (Tsingke) for product validation. Primer sequences are detailed in table S4.

### Tn5-FISH

FISH was performed as previously described (*28*). Unique nonrepetitive sequences flanking the chromosomal breakpoints on 1q25 and 22q13 were amplified by PCR (∼25 kb spanning each locus). PCR products were purified (DNA Cleanup kit, Vazyme), followed by tagmentation with Tn5 transposase (Vazyme) to generate fragments < 500 bp. Tagmented DNA was amplified using fluorescence-conjugated primers: Alexa Fluor 488 (chromosome 22) and Alexa Fluor 568 (chromosome 1). Probes were purified (DNA Cleanup kit, Vazyme), and primer sequences are detailed in table S7.

GM12878 cells or CD138^+^/CD138^−^ cells isolated from primary myeloma patient samples were pelleted (500*g*, 5 min, RT), washed with PBS, and fixed in Carnoy’s fixative (methanol:acetic acid, 3:1). Fixed cells were immobilized on poly-l-lysine–coated slides, air-dried, and permeabilized (0.5% Triton X-100, 20% glycerol in PBS, 30 min, RT). Hybridization was performed with 10 ng each of green (22q13) and red (1q25) probes in 20 μl of hybridization buffer (50% deionized formamide, 2× SSC, 10% dextran sulfate). Slides were denatured (75°C, 5 min), hybridized overnight (37°C), and washed sequentially in stringent buffer (0.4% NP-40, 2× SSC, and 10% formamide; 73°C, 2 min; RT, 5 min) followed by PBS. Nuclei were counterstained with 4′,6-diamidino-2-phenylindole (BIORIGIN) and imaged using a Nikon AX confocal microscope (100× oil immersion objective). Image analysis was conducted using ImageJ (v1.54f, National Institutes of Health).

### Optical genome mapping

High–molecular weight DNA was isolated from bone marrow aspirates using the Bionano Prep SP Frozen Human BMA DNA Isolation Kit (Bionano Genomics) and fluorescently labeled with the DLS DNA Labeling Kit (Bionano Genomics). Labeled DNA molecules were linearized on Saphyr G2.3 chips and imaged using the Saphyr instrument (Gen 2, Bionano Genomics) for structural variant analysis.

### 4C-seq

Briefly, 10^7^ formaldehyde-crosslinked cells (2%, 10 min, RT) were lysed in hypotonic buffer (10 mM tris-HCl, 10 mM NaCl, 0.2% NP-40, and protease inhibitor), and nuclei were digested sequentially with 200 U of DpnII (NEB; 37°C, overnight) followed by T4 DNA ligase–mediated intramolecular ligation (4000 U, 16°C, overnight) in 1× ligation buffer containing 1% Triton X-100. Crosslink reversal and DNA purification were achieved through proteinase K digestion (65°C, overnight), phenol-chloroform extraction, and ethanol precipitation.

Viewpoint-specific digestion was performed using NlaIII or CviQI (NEB), followed by secondary ligation and template quantification (Qubit dsDNA BR Assay Kit, Thermo Fisher Scientific). Quadruplicate PCR amplifications per sample were conducted using the Expand Long Template PCR System (Roche; 16 cycles) with viewpoint-specific primers (primers in table S8). Primary amplicons were size-selected using Hieff NGS DNA selection beads (Yeasen) and subjected to nested PCR (20 cycles) with Illumina adapter–containing universal primers (94°C/10 s, 60°C/1 min, 68°C/3 min). Final libraries were purified (QIAquick PCR Purification Kit, QIAGEN), quality-controlled via agarose gel electrophoresis and NanoDrop spectrophotometry (Thermo Fisher Scientific), and sequenced as 150-bp paired-end reads on the Illumina NovaSeq 6000 platform.

### 3C assays

3C-qPCR was performed following established protocols (*30*) with minor modifications. Briefly, crosslinking was performed on 2 × 10^6^ LP-1 cells [wild-type and t(1;22)(q25;q13)-engineered variants] using 1% formaldehyde for 10 min at 25°C. Reactions were quenched with 0.13 M glycine (5 min), followed by two PBS washes. Cell pellets were resuspended in ice-cold lysis buffer (10 mM tris-HCl, 10 mM NaCl, 0.2% NP-40, and protease inhibitor cocktail) and incubated on ice for 20 min. Nuclei were pelleted (500*g*, 5 min) and resuspended in 1.2× DpnII buffer (NEB) containing 0.3% SDS. After incubation at 37°C for 1 hour with agitation, Triton X-100 was added to 2.5% final concentration for an additional 1 hour. Chromatin was digested with 200 U of DpnII (NEB) overnight at 37°C, followed by heat inactivation (65°C, 20 min).

Digested chromatin was diluted to 1 to 2 ng/μl in T4 DNA ligase buffer (NEB) with 1% Triton X-100 and ligated overnight at 16°C with 4000 U of T4 DNA ligase. After RNase A treatment (0.5 μg/ml, 37°C, 30 min), samples were de-crosslinked overnight at 65°C with 20 μl of proteinase K (10 mg/ml). DNA was purified via phenol-chloroform extraction and ethanol precipitation and quantified using a Qubit dsDNA BR Assay Kit (Thermo Fisher Scientific).

Spatial interaction frequencies were determined using a primer-walking strategy by Taq Pro Universal SYBR qPCR Master Mix (Vazyme) on a QuantStudio 3 Real-Time PCR System (Thermo Fisher Scientific). Primer pairs comprised a fixed anchor primer (P0) at the *CBX7* promoter and eight test primers (P1 to P8) spanning the hijacked enhancer region at ∼1.4-kb intervals (range > 1 kb), with P5 specifically interrogating the enhancer core. Primer specificity was validated through in silico prediction (NCBI Primer-BLAST) and dissociation curve analysis. Glyceraldehyde phosphate dehydrogenase–normalized interaction frequencies were calculated, with primer sequences detailed in table S9.

### Real-time reverse transcription quantitative PCR (RT-qPCR)

Total RNA was isolated using TRIzol reagent (TransGen Biotech), followed by cDNA synthesis with the Reverse Transcription System (Vazyme). Quantitative analysis of gene expression was performed on a QuantStudio 3 Real-Time PCR System (Thermo Fisher Scientific) using Taq Pro Universal SYBR qPCR Master Mix (Vazyme), with mRNA level of β-actin serving as the endogenous control. Primer sequences are provided in table S10.

### Luciferase reporter assay

Enhancer regions amplified from LP-1 genomic DNA (2× SuperNova PCR Mix, GenStar; primers in table S11) were cloned into the pGL4.23[luc2/minP] vector (Promega) via SacI/HindIII (NEB) restriction sites and T4 DNA ligase (NEB), with constructs verified by sequencing. LP-1 cells (5 × 10^5^) were co-electroporated with 0.5 μg of enhancer-pGL4.23 and 5 ng of pRL-TK (Renilla control) using the Lonza 4D Nucleofector SF Kit (DS-150 program). Luciferase activity was measured 48 hours posttransfection via the Dual-Luciferase Reporter Assay System (Promega), with firefly signals normalized to Renilla values.

### CRISPR-Cas9–mediated translocation engineering

A dual sgRNA/Cas9 coexpression system was engineered using a modified LentiCRISPRv2 backbone (Addgene). sgRNAs targeting t(1;22)(q25;q13) breakpoints were designed by integrating LRS and PCR data, with optimization using CRISPOR and CRISPick algorithms (table S12). Top-ranked sgRNAs were cloned into BsmBI-v2-digested (NEB) vectors via T4 DNA ligase (NEB), followed by Stbl3-competent cell (TransGen Biotech) transformation and plasmid purification (TIANGEN EndoFree Mini Plasmid Kit). Validated constructs were packaged into lentivirus via human embryonic kidney 293T cotransfection (Lipofectamine 3000, Thermo Fisher Scientific) with pMD2.G/psPAX2, concentrated using Amicon Ultra-15 filters (Sigma-Aldrich), and transduced into LP-1 cells.

Puromycin-selected cells (2 μg/ml, 6 days) were clonally isolated in 96-well plates. Translocation screening used breakpoint-flanking primers (table S12) using Taq polymerase (GenStar), with 1100-bp amplicons indicating successful rearrangements. Positive clones were expanded, and genomic DNA (QIAGEN Blood and Tissue Kit) was subjected to secondary PCR amplification. Unique breakpoint junctions were confirmed by Sanger sequencing (Tsingke), with clonal independence verified through distinct sequencing profiles.

### CCK-8 assay

Cell proliferation was assessed using the CCK-8 kit (TransGen Biotech) according to the manufacturer’s protocol. Absorbance measurements were recorded at 24-hour intervals.

### Colony formation assay

Cells were seeded in methylcellulose medium (STEMCELL Technologies) and incubated for 12 days. Colony-forming units were quantified microscopically, with colony counts normalized to initial cell seeding density.

### Patients’ survival analysis

Consecutive cases of patients with myeloma diagnosed at Peking University People’s Hospital and Fu Xing Hospital of Capital Medical University from January 2019 to December 2024 were included. Patients diagnosed with myeloma met the International Myeloma Working Group diagnostic criteria 2014 (*47*). FISH of bone marrow was performed at baseline to detect chromosomal abnormalities using CD138-purified plasma cells isolated by magnetic-activated cell sorting (MACS), as previously described (*48*). All patients were analyzed for *IGH* translocation using gene locus-specific probes. If an *IGH* translocation was identified, dual-color and dual-fusion translocation probes such as *IGH*-*FGFR3*, *IGH*-*MAF*, and *IGH*-*CCND1* were used to detect t(4;14)(p16;q32), t(14;16)(q32;q23), and t(11;14)(q13;q32). Survival functions were estimated using the Kaplan-Meier method and compared using the log-rank test. PFS and OS were defined as the interval from diagnosis to disease progression or death in the PFS model and death from any cause in the survival model. Patients were censored at last contact. The last follow-up time was December 2024.

### SV analysis

For the CLR mode of SMRT-seq, reads were initially aligned to hg38 using pbmm2 (v.1.13.1) with parameters “pbmm2 align --median-filter --preset SUBREAD.” SV signatures were detected using pbsv (v.2.9.0) with default parameters, and SVs were subsequently called using the command “pbsv call -m 50.” For HiFi mode, SMRT reads were aligned to hg38 using pbmm2 with parameters “pbmm2 align --preset HIFI.” SV signatures were detected using pbsv with the parameters “pbsv discover --hifi,” followed by variant calling with “pbsv call -m 50 --hifi.” SVs were visualized using Circos (v.0.69.8), IGV (v.2.17.4), and Ribbon (v.1.3.0) (*49*) with default parameters. Random sequences were generated with bedtools (v.2.30.0) (*50*) using the “bedtools shuffle” function.

A nonredundant set of discovered SVs across 13 myeloma samples was constructed using a merging strategy. The IM9 cell line was used as the initial set, and unique SVs from each additional sample were added. Merging was conducted independently for each variant type, and the resultant sets were concatenated to produce a final nonredundant SV set. SVs were considered unique if the distance between their breakpoints and those in the myeloma sample set exceeded 500 bp using “bedtools intersect.”

The distribution of SVs was analyzed by calculating the distance from SV breakpoints to chromosomal termini and TSSs. These distributions were visualized using ggplot2 (v.3.5.1). The proportion of SVs within the myeloma sample set was calculated using the “bedtools annotate -counts” function. The potential pathogenetic impact of SVs in myeloma was assessed using phenosv.py (*22*) with the parameters “--model PhenoSV --noncoding tad”. The dominant translocations in each myeloma sample were evaluated using AD/DP values from SMRT-seq data and visualized through the CMplot package (v.4.5.1). Sequence similarity near breakpoints of t(4;10;18) was assessed using NCBI BLAST.

### Hi-C data analysis

Paired-end reads were initially trimmed using Trimmomatic (v.0.39) (*51*) to remove adapter sequences and low-quality bases. For in situ Hi-C data, the trimmed reads were aligned to hg38 and subsequently processed into normalized contact matrices through the HiC-Pro pipeline (v.3.1.0) (*52*). Default settings were used to remove duplicate reads, assign reads to restriction fragments GATCGATC, filter invalid pairs, and generate both raw and ICE-normalized contact matrices at various resolutions. In addition, hic files were generated using hicpro2juicebox. Micro-C XL contact matrices were produced using the Dovetail Genomics pipeline (https://micro-c.readthedocs.io/en/latest/). Similar to the Hi-C analysis, Trimmomatic (v.0.39) (*51*) was used to remove adapters and low-quality reads. The read pairs were then mapped to hg38 using BWA-MEM (v.0.7.17) (*53*). Subsequently, PCR duplicates and reads with low mapping quality (<40) were filtered out using pairtools (v.1.0.3). Juicertools (v.1.22.01) was used to convert pairs files into hic files, and mcool files with multiple fixed bin sizes were subsequently created using hic2cool (v.0.8.3) and balanced using cooler (v.0.9.3) (*54*). Hierarchical clustering of KMS11, MM.1S, LP-1, RPMI8226, U266, PT1, PT2, and PT3 based on raw Hi-C matrices with 500-kb resolution was conducted using HiCExplorer (v.3.7.2) (*55*) with the parameter “hicCorrelate --method pearson --log1p.” Insulation scores were calculated at 50-kb resolution using the Perl script matrix2insulation.pl of cworld (*15*) with parameters “--is 500000 --ids 250000 --im mean --bmoe 0 --nt 0.1.” Insulation scores at SV breakpoints were visualized using deepTools (v.3.5.2) (*56*) with default parameters. Hi-C matrix visualization of translocations was performed using Juicer (1.11.08) (*57*) and EagleC (v.0.1.8) (*25*) with the parameters “plot-interSVs --balance-type Raw --cool-uri contact.mcool::resolutions/1000000.”

### CUT&Tag data analysis

Sequencing reads were preprocessed using fastp (v.0.23.2) (*58*) with the “--detect_adapter_for_pe” option to remove adapter sequences. Subsequently, the trimmed reads were aligned to hg38 using Bowtie2 (v.2.2.5) (*59*) with “--very-sensitive -X 1000” parameters to ensure high alignment sensitivity. PCR duplicates were identified and removed using the Picard (v.2.27.5) MarkDuplicates with “REMOVE_DUPLICATES = true VALIDATION_STRINGENCY = LENIENT” settings. For peak calling of histone marks, MACS2 (v.2.2.7.1) was used with the “-g hs -B -q 0.05 -f BAMPE” option. Last, bigWig files were generated from BAM files using deeptools (v.3.5.2) (*56*) with the “bamCoverage --normalizeUsing RPGC” option to enable normalization for downstream visualization. Enhancers were defined using H3K27ac peaks derived from CUT&Tag data.

### RNA-seq data analysis

For RNA-seq data processing, raw sequencing reads were initially trimmed using fastp (v.0.23.2) (*58*) with the --detect_adapter_for_pe option to remove adapter sequences. The trimmed reads were then aligned to hg38 using HISAT2 (v.2.2.1) (*60*) with default parameters. Transcript abundance was quantified in terms of TPM using StringTie (v2.2.3) (*61*) with the following parameters: “stringtie -G gencode.v40.annotation.gtf -e -A output.tpm input.bam.” For samples with replicates, the average TPM values were calculated to represent expression levels. Last, bigWig files were generated from BAM files using deepTools (v.3.5.2) (*56*) with the “bamCoverage --normalizeUsing BPM” option for downstream visualization. RNA-seq data from HD1, a normal plasma cell reference, were processed using the same pipeline, and gene expression was transformed as log_2_(TPM + 1) for comparison with the corresponding translocation-positive samples.

### ATAC-seq data analysis

Raw ATAC-seq reads were initially processed using fastp (v.0.23.2) (*58*) with the --detect_adapter_for_pe option to remove adapter sequences. Subsequent alignment to hg38 was performed using Bowtie2 (v.2.2.5) (*59*) with the parameters “--very-sensitive -X 2000.” PCR duplicates were identified and removed using the Picard (v.2.27.5) MarkDuplicates with the settings REMOVE_DUPLICATES = true VALIDATION_STRINGENCY = LENIENT. To correct Tn5 transposase insertion bias, aligned reads were shifted +4 bp on the “+” strand and −5 bp on the “−” strand before peak calling. Peak detection was conducted using MACS2 (v.2.2.7.1) with “--shift -75 --extsize 150 --nomodel --SPMR -g hs -B -q 0.05 -f BAMPE --keep-dup all” options. BigWig files were generated from BAM files using deepTools (v.3.5.2) (*56*) with the bamCoverage --normalizeUsing RPGC option for downstream visualization.

### 4C-seq data analysis

4C-seq reads were processed using the 4C pipeline, available at https://github.com/deLaatLab/pipe4C. Paired-end sequencing reads were trimmed using fastp (v.0.23.2) (*58*) with the --detect_adapter_for_pe option to remove adapters. The pipe4C.R script was used to map and analyze chromatin interactions, focusing on promoter regions of the target genes, thereby providing a high-resolution view of long-range chromatin contacts.

### TransFinder pipeline

Translocations were called independently of SMRT-seq data using pbsv and from Hi-C contact matrices using EagleC (*25*). Events supported by both datasets were retained as high-confidence translocations when the corresponding breakpoint coordinates were within 240 kb. For these events, SMRT-seq breakpoint-spanning reads were used to define base pair breakpoints and fragment orientation. For each high-confidence translocation, CNV-normalized Hi-C contact maps were locally reconstructed around the breakpoint regions to model the rearranged 3D chromatin architecture. Translocation-induced neo-loops were identified with NeoLoopFinder (*62*). Neo-loops were annotated as E-P neo-loops when one anchor overlapped a gene promoter (±1 kb of the TSS) and the other overlapped an H3K27ac-defined enhancer (ChIP-seq/CUT&Tag). Putative target genes were prioritized using RNA-seq, retaining genes with TPM > 10; when multiple genes were linked to the same neo-loop, the gene with the highest TPM was carried forward. Neo-loops and associated regulatory features were visualized using NeoLoopFinder (*62*).

### SVs called from OGM

Approximately 1.5 Tbp of OGM data was collected per myeloma sample. Standard quality control parameters, including total DNA from molecules ≥ 150 kbp, N50 for molecules ≥ 150 kbp, map rate, effective coverage, and average label density (labels per 100 kbp), were evaluated according to the manufacturer’s guidelines. The OGM data were analyzed using the rare variant analysis (RVA) pipeline and visualized in Bionano Access. Default settings were used for the initial RVA. SV calls were annotated against the Bionano human control SV database. To exclude SVs observed in the control population, the maximum control database frequency was set to 0%.

### DepMap analysis

Chronos CRISPR gene effect scores, common-essential gene annotations, model metadata, and RNA-seq expression data were obtained from DepMap. Analyses were restricted to the 18 genes nominated by TransFinder. For each gene, the distribution of Chronos gene effect scores across DepMap models was examined, and Spearman’s rank correlation between RNA-seq expression and Chronos gene effect scores was calculated using models with available data for both variables.

### Statistical analysis

All box plots were generated using R (v.4.4.0) and are presented as medians and interquartile ranges. Validation experiments were performed independently three or four times, and data are presented as mean ± SD. Statistical analyses were performed using overall log-rank tests, Wilcoxon rank sum tests, Spearman correlation analysis, two-tailed unpaired Student’s *t* tests, one-way analysis of variance (ANOVA) with Dunnett’s test versus PT3 or CTR, or two-way ANOVA. Where applicable, *P* values were adjusted for multiple comparisons using the Benjamini-Hochberg method. A *P* value of less than 0.05 was considered statistically significant. Significance levels are indicated as *P < 0.05, **P < 0.01, ***P < 0.001, and ****P < 0.0001; ns indicates not significant.
