# Supplementary material for: Translocation-induced enhancer rewiring reveals cryptic oncogenic circuits in multiple myeloma
Source: Sci Adv. 2026 Oct 7;12(41):eaeg9610. doi: 10.1126/sciadv.aeg9610 (PMC13644794; doi:10.1126/sciadv.aeg9610)
Supplement: Supplementary file 1 [file sciadv.aeg9610_sm.pdf]

Science Advances
AAAS

Supplementary Materials for

Translocation-induced enhancer rewiring reveals cryptic oncogenic circuits in multiple myeloma

Enrun Zheng *et al.*

Corresponding author: Jichuan Wang, jichuan.wang@outlook.com; Jin Lu, lujin@pku.edu.cn; Hebing Chen, chb-1012@163.com; Luyang Sun, luyang_sun@hsc.pku.edu.cn

Sci. Adv. **12**, eaeg9610 (2026)
DOI: 10.1126/sciadv.aeg9610

The PDF file includes:

Figs. S1 to S9
Legends for tables S1 to S12

Other Supplementary Material for this manuscript includes the following:

Tables S1 to S12

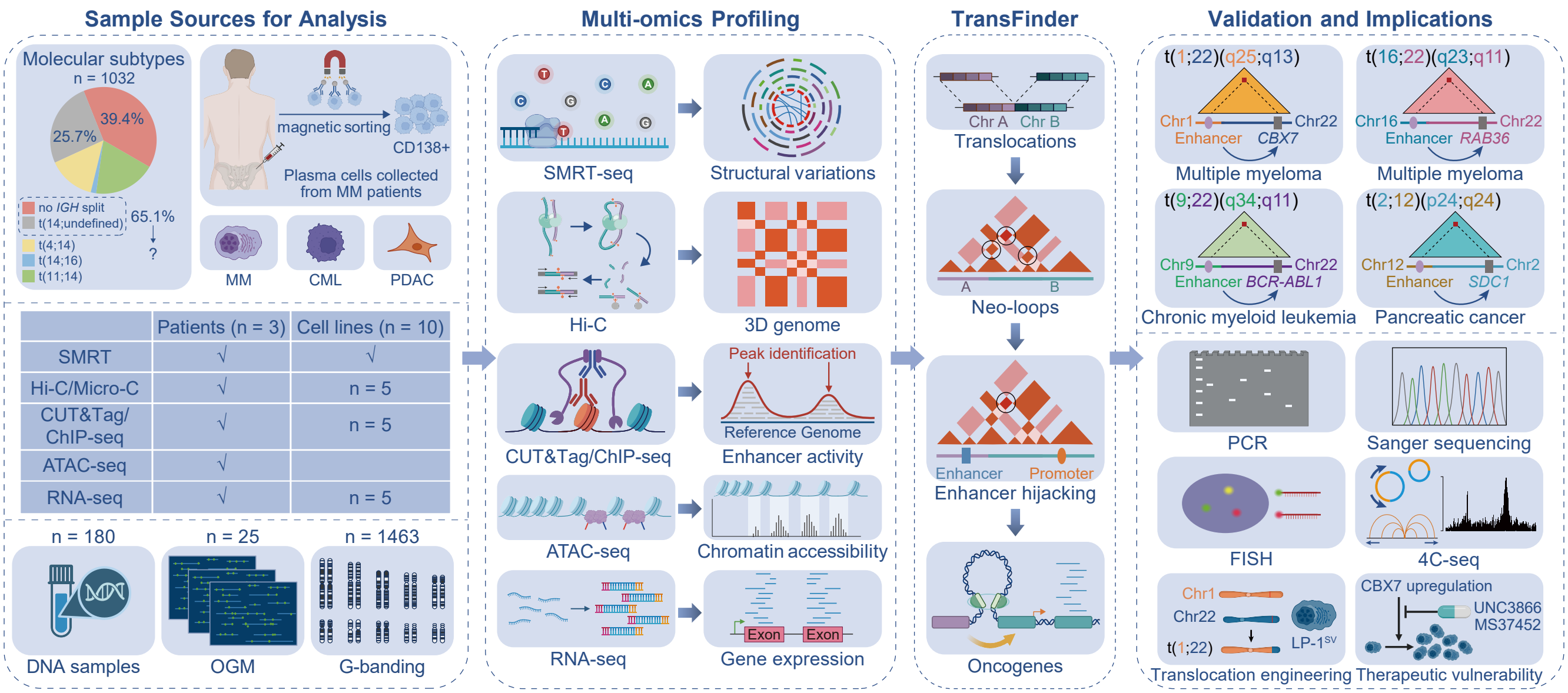

Fig. S1. Integrated multi-omics profiling identifies translocation-driven enhancer hijacking across malignancies. The schematic outlines the study workflow, from sample collection and multi-omics profiling to the identification and validation of translocation-induced enhancer-hijacking events. Created in BioRender. Li, Q. (2026) https://BioRender.com/z96m864.

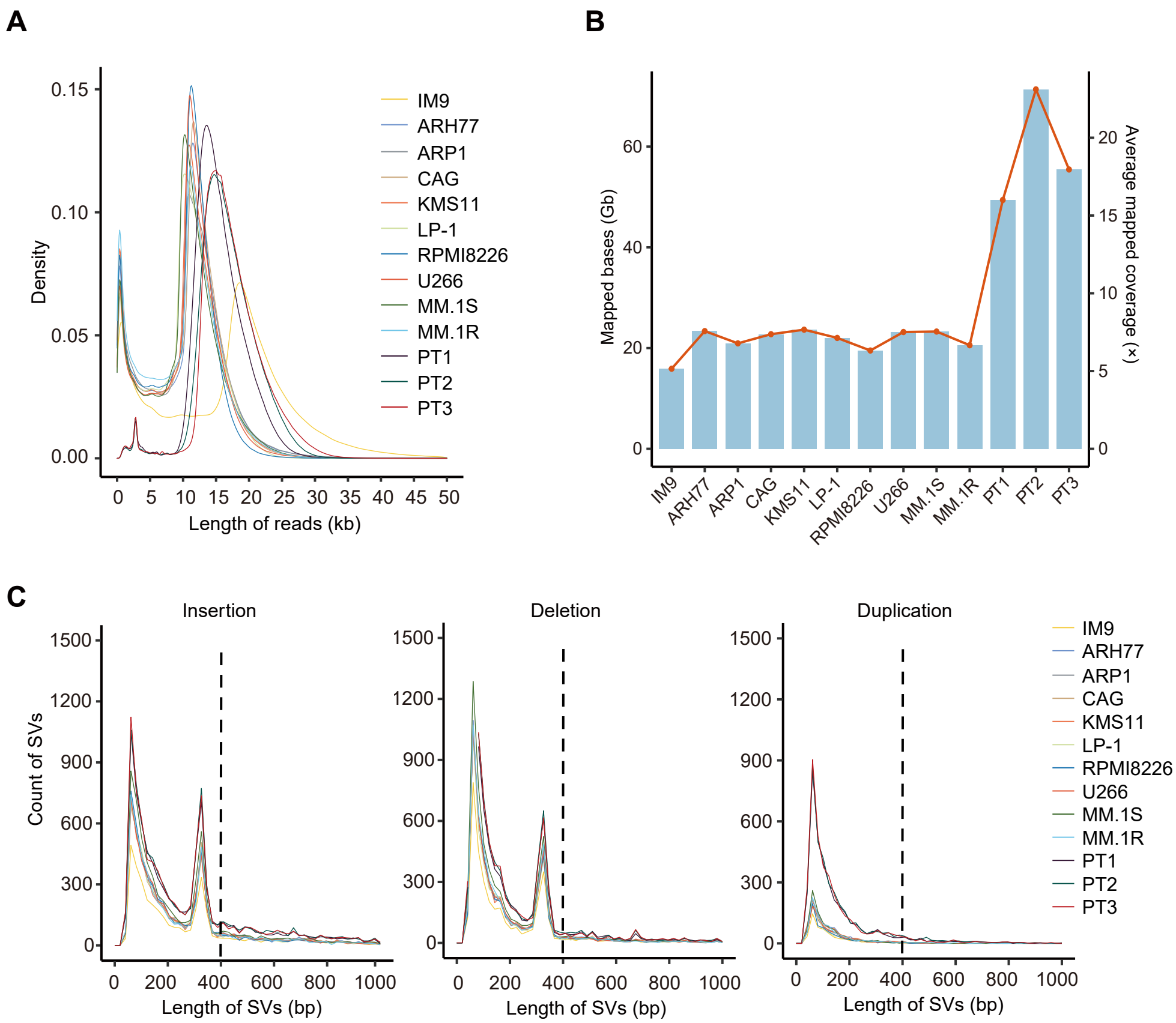

Fig. S2. Long-read sequencing profiles structural variation across multiple myeloma samples and cell lines. (**A**) Density plot showing the distribution of subread lengths derived from myeloma samples. (**B**) Bar plots indicate the total mapped bases (Gb) generated for each myeloma sample, while the line plot shows the corresponding average mapped genome coverage (×). (**C**) A frequency polygon plot showing the distribution of SV lengths across myeloma specimens.

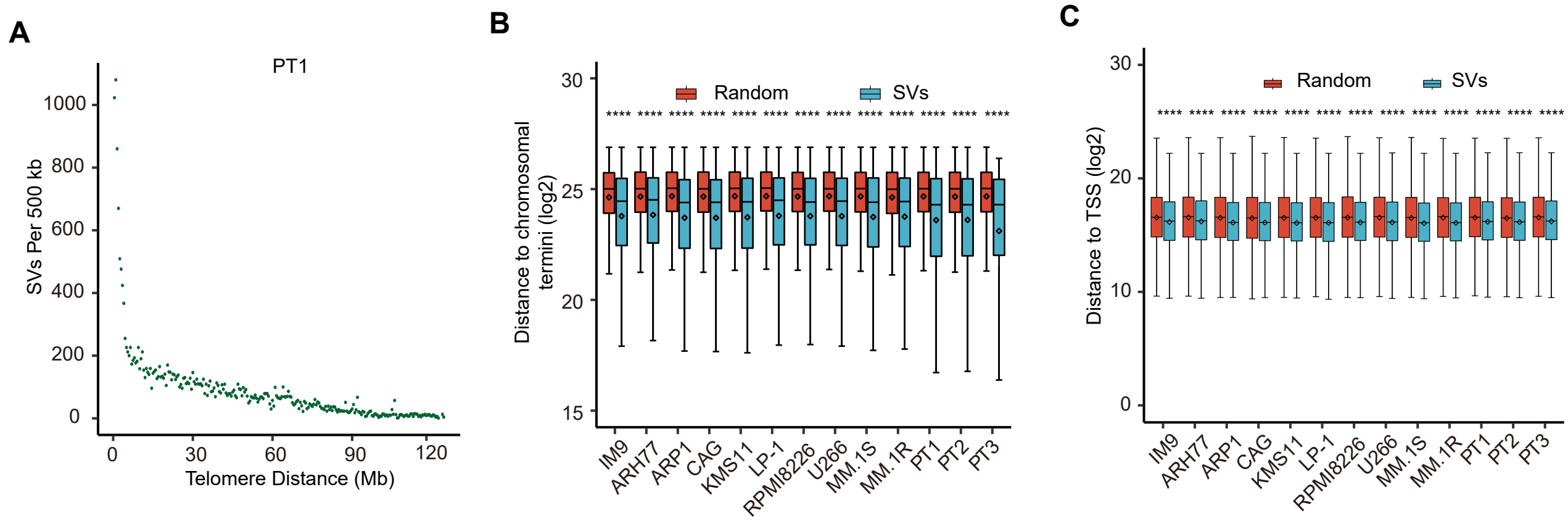

Fig. S3. Structural variants are enriched near chromosome ends and transcription start sites. (**A**) For each identified SV in the PT1 sample, the genomic proximity to chromosomal termini was quantified and segregated into discrete bins of 500 kb, allowing for a precise evaluation of SV localization relative to chromosomal termini. (**B**) Box plots comparing the distances of random sequences and SVs from chromosomal termini across 13 myeloma samples. (**C**) Box plots comparing the distances of random sequences and SVs from transcription start sites (TSSs) across 13 myeloma samples. For (B) and (C), within-sample comparisons were performed using two-sided Wilcoxon rank-sum tests, and P values were adjusted across the 13 comparisons within each panel using the Benjamini–Hochberg method. Center lines indicate medians, boxes indicate interquartile ranges, whiskers extend to the most extreme values within 1.5 times the interquartile range, and diamonds indicate means. $^*P < 0.05$, $^{**}P < 0.01$, $^{***}P < 0.001$, $^{****}P < 0.0001$; ns, not significant.

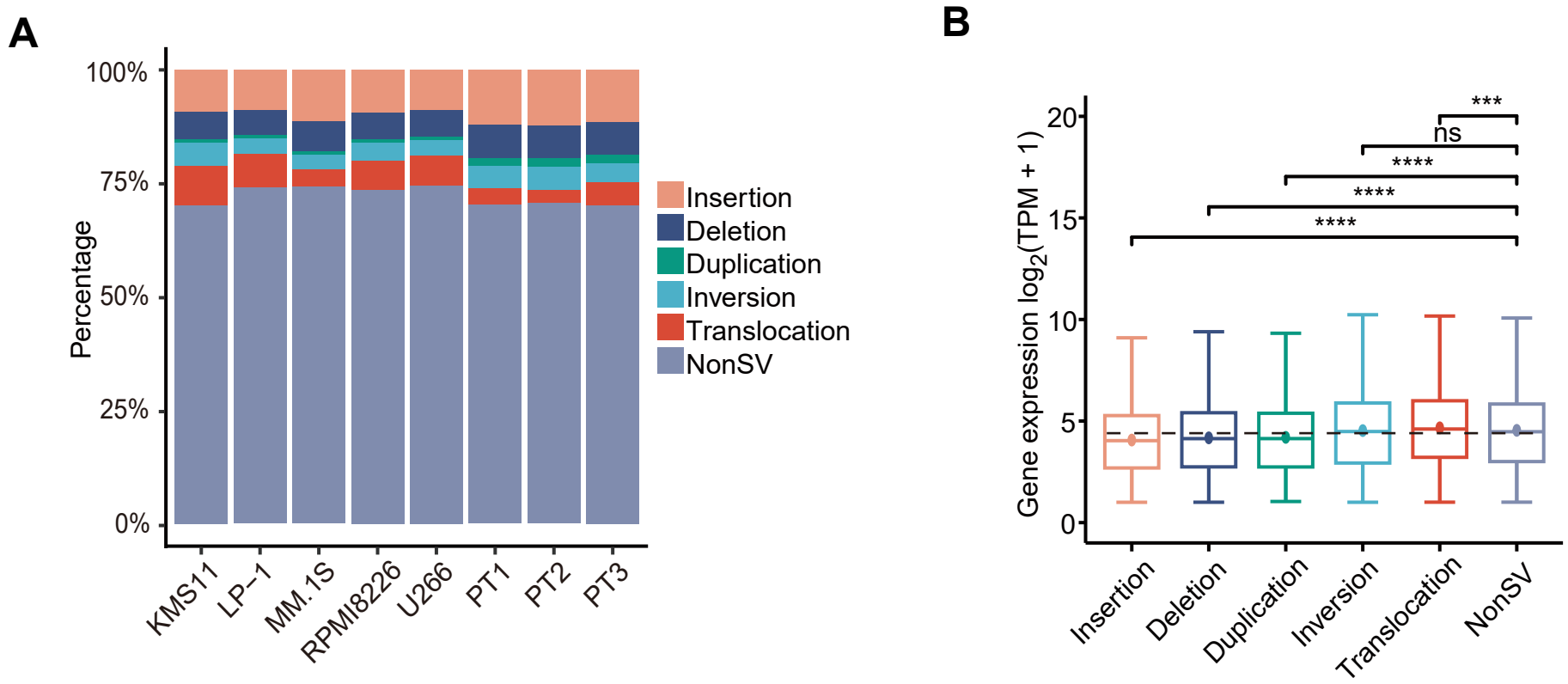

Fig. S4. Structural variant classes show distinct gene associations and transcriptional profiles in multiple myeloma. (**A**) The proportion of genes affected by different types of SVs and those unaffected by SVs in myeloma samples. (**B**) The expression of genes in different SV-associated categories compared to genes not associated with SVs in myeloma samples including KMS11, LP-1, MM.1S, RPMI8226, U266, PT1, PT2, and PT3. Statistical significance was determined using the Wilcoxon rank-sum test with Benjamini-Hochberg method. **P* < 0.05, ***P* < 0.01, ****P* < 0.001, *****P* < 0.0001; ns, not significant.

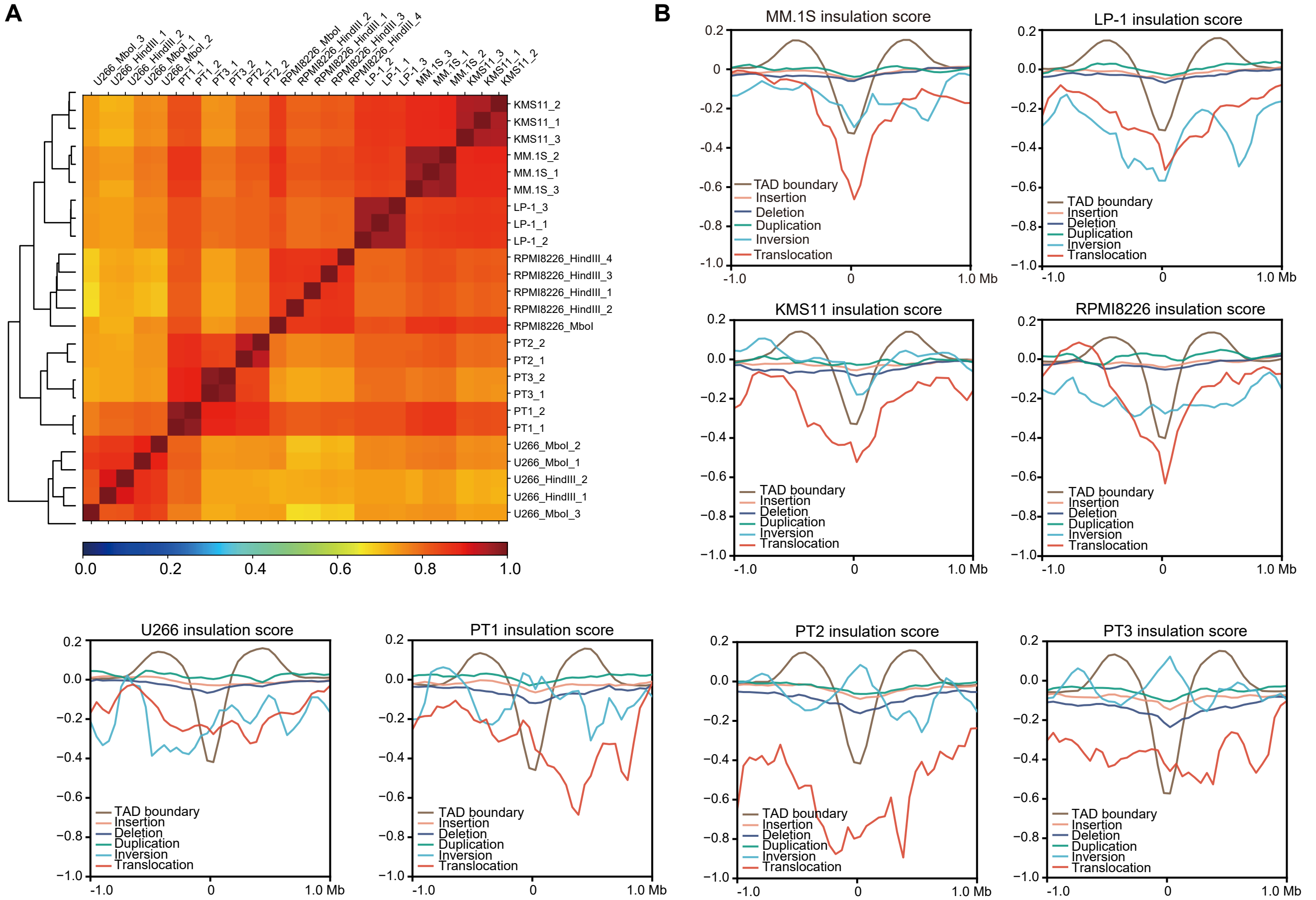

Fig. S5. Chromosome translocation correlates with 3D genome reorganization in myeloma cells. (**A**) Quality control of Hi-C data by calculating the correlation coefficients across multiple Hi-C contact matrices at a 500 kb resolution. (**B**) Insulation scores were calculated within 1 Mb region flanking breakpoint sites across various SVs and TAD boundaries in MM.1S, KMS11, LP-1, RPMI8226, U266, PT1, PT2, and PT3.

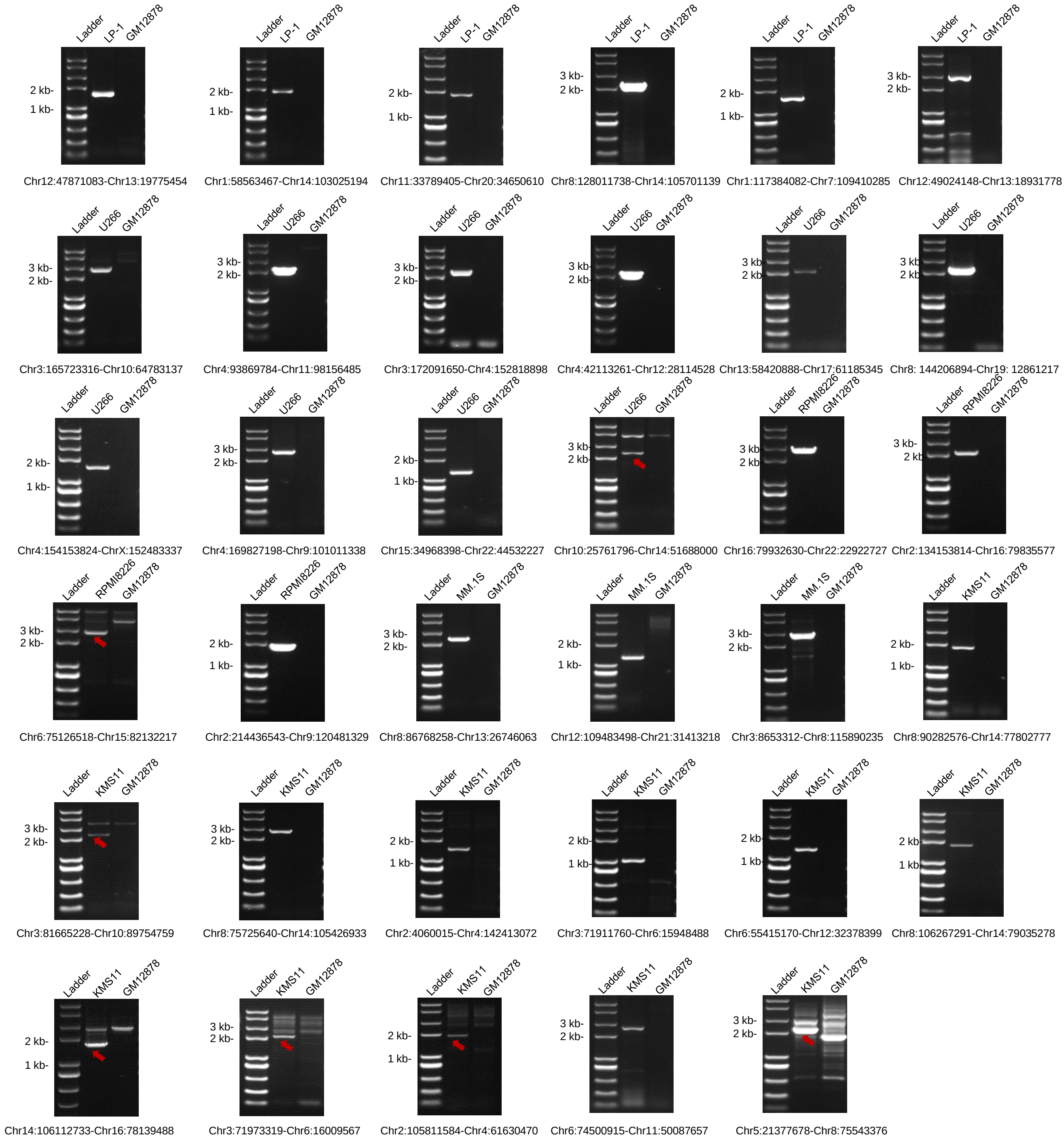

Fig. S6. Validation of translocation breakpoints by junction-spanning PCR. Translocations identified by TransFinder in myeloma cell lines were validated using junction-spanning PCR.

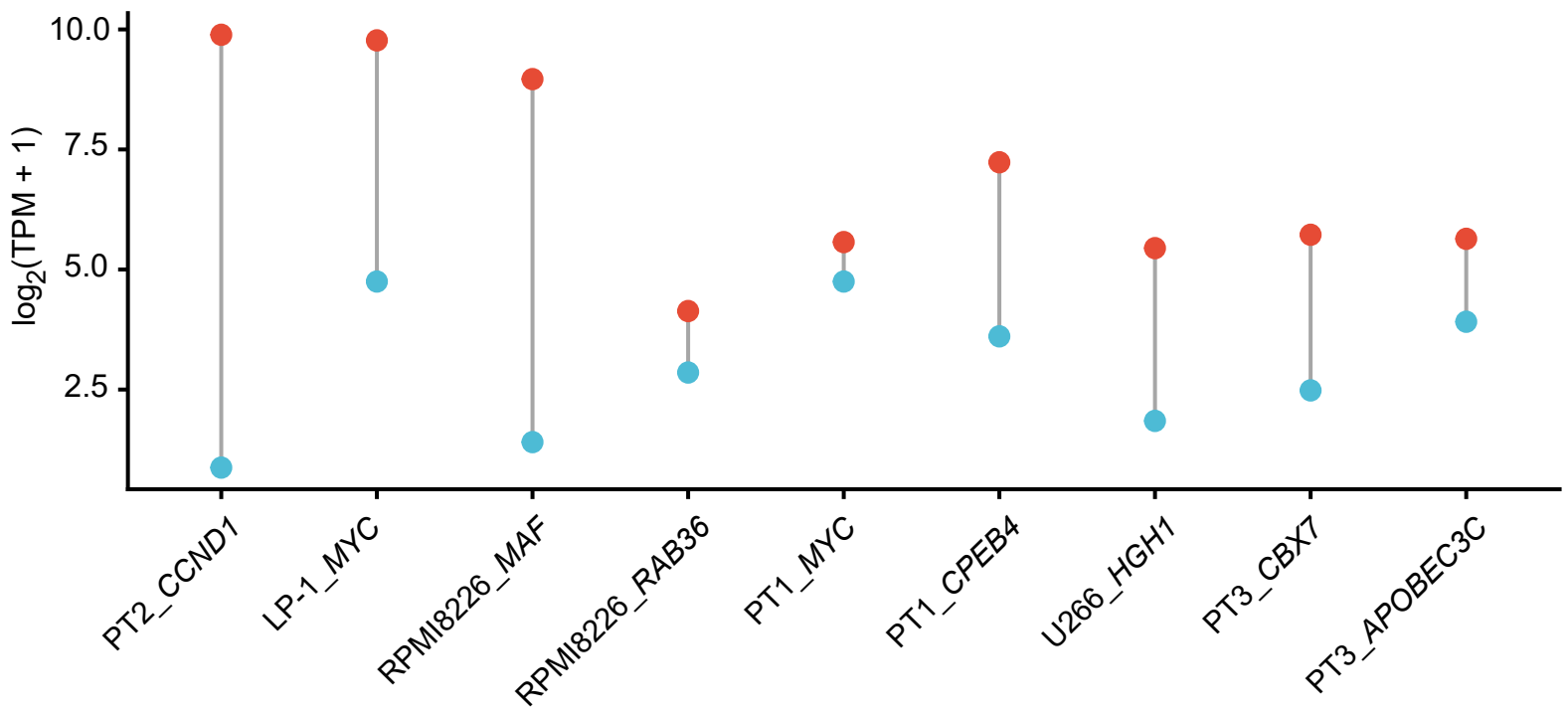

Fig. S7. Comparison of RNA-seq expression for TransFinder-nominated myeloma target genes in translocation-positive samples and the HD1 normal plasma-cell reference. Expression is shown as $\log_2$(TPM + 1). Blue dots indicate HD1, and red dots indicate the corresponding translocation-positive samples. Lines connect the paired expression values for each nominated gene.

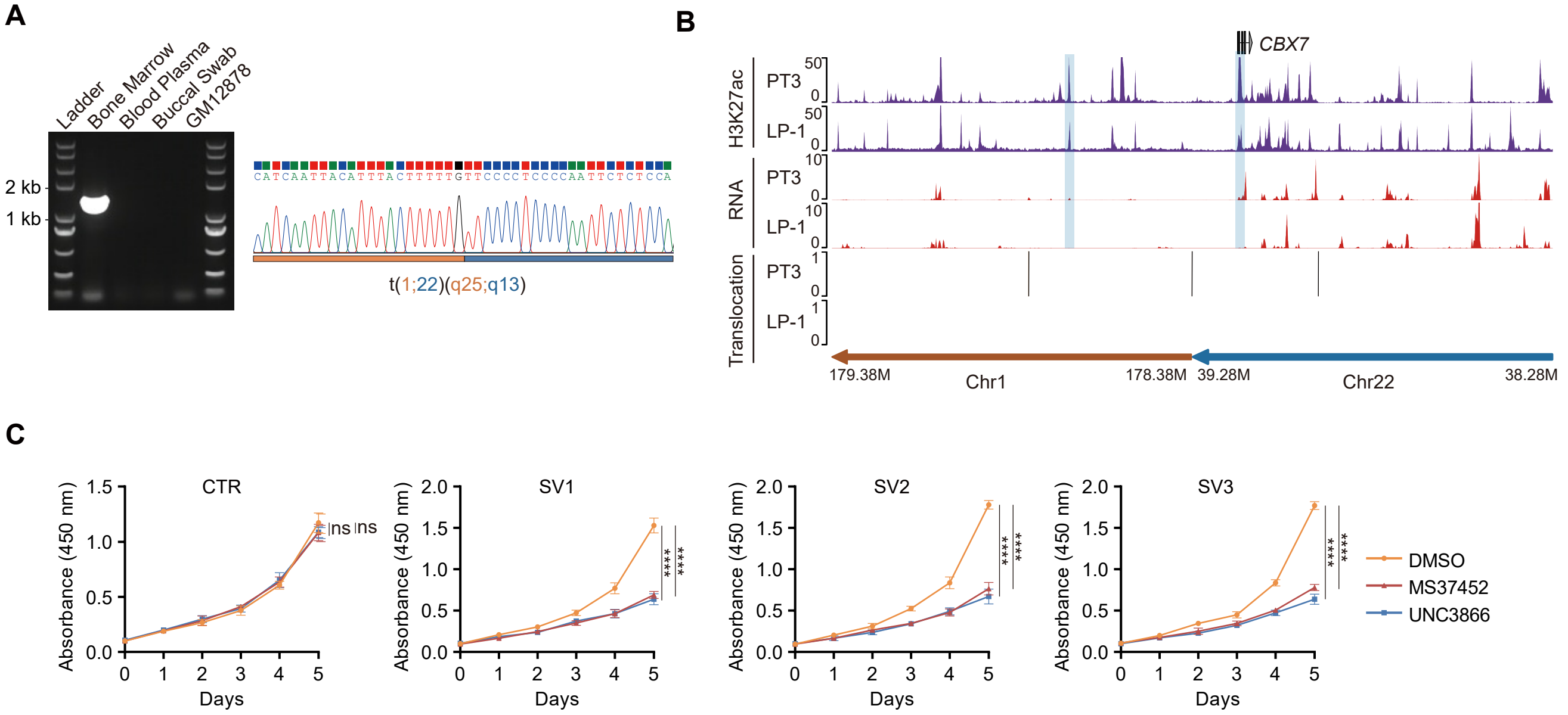

Fig. S8. Engineering t(1;22) increases the sensitivity of myeloma cells to CBX7 inhibition. (**A**) PCR results for t(1;22)(q25;q13) in bone marrow, blood plasma, and buccal swabs from a patient-derived sample. Sanger sequencing verified the presence of t(1;22)(q25;q13). (**B**) Integrated analyses using ChIP-seq/CUT&Tag for H3K27ac, RNA-seq and SMRT-seq elucidating translocations and enhancers surrounding PT3-derived t(1;22)(q25;q13) breakpoint in PT3 and LP-1 cells. Enhancers and promoters are highlighted with light blue bars. (**C**) Assessment of cell proliferation by CCK-8 assays in CTR, SV1, SV2, and SV3 treated with DMSO, 10 µM MS37452, or 10 µM UNC3866 (n = 4). *P* values were calculated using two-way ANOVA. **P* < 0.05, ***P* < 0.01, ****P* < 0.001, *****P* < 0.0001; ns, not significant.

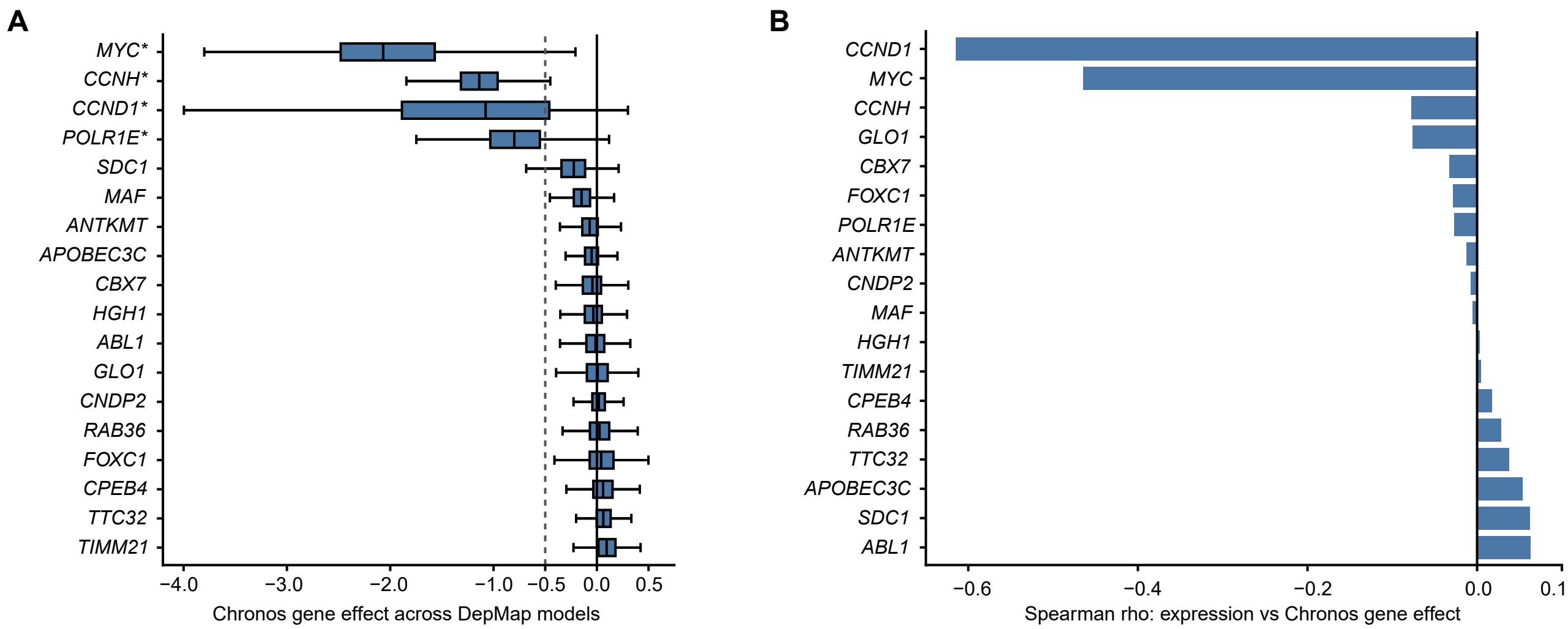

Fig. S9. DepMap-based functional assessment of TransFinder-nominated genes. (A) Pan-cancer distributions of Chronos CRISPR gene-effect scores for the 18 TransFinder-nominated genes across DepMap cell-line models. Boxes indicate the median and interquartile range; whiskers extend to 1.5 times the interquartile range, with outliers omitted. The dashed vertical line marks the −0.5 dependency threshold. Asterisks indicate genes classified by DepMap as common essentials. Lower Chronos scores indicate stronger dependency. **(B)** Correlations between gene expression and CRISPR dependency for the 18 TransFinder-nominated genes across DepMap models. Spearman correlation coefficients were calculated between RNA expression and Chronos gene-effect scores. Negative coefficients indicate that higher expression is associated with stronger dependency.

Table S1. IGH rearrangement status and survival outcomes in 1,032 patients with multiple myeloma
Table S2. Summary of clinical and molecular features of PT1, PT2, and PT3
Table S3. List of 48,864 non-redundant structural variants in multiple myeloma
Table S4. 42 experiment-confirmed high-confidence inter-chromosomal translocations identified by TransFinder
Table S5. 13 high-confidence translocations supported by G-banding
Table S6. Translocation-induced enhancer-hijacking neo-loops in myeloma cells
Table S7. Primer sequences used for Tn5-FISH analysis
Table S8. DNA oligos for viewpoint-specific primers in 4C-seq validation
Table S9. Primer sequences for 3C-qPCR-based relative interaction frequency quantification
Table S10. qPCR primer sequences for gene expression quantification
Table S11. DNA oligos for hijacked enhancers cloning in luciferase reporter assay
Table S12. Primer and sgRNA sequences used for generation of t(1;22)(q25;q13)
